# Dual Roles of *OsGH3.2* in Modulating Rice Root Morphology and Affecting Arbuscular Mycorrhizal Symbiosis

**DOI:** 10.3389/fpls.2022.853435

**Published:** 2022-04-11

**Authors:** Cheng-Chen Liu, Ying-Na Liu, Jian-Fei Cheng, Rui Guo, Li Tian, Bin Wang

**Affiliations:** State Key Laboratory of Pharmaceutical Biotechnology, School of Life Sciences, Nanjing University, Nanjing, China

**Keywords:** rice (*Oryza sativa*), arbuscular mycorrhiza (AM), *OsGH3.2*, auxin, root architecture, cortical cell length, arbuscular size

## Abstract

Several angiosperm *GRETCHEN HAGEN 3* (*GH3*) genes, including tomato *SlGH3.4* and rice *OsGH3.2* are induced during arbuscular mycorrhizal (AM) symbiosis, but their functions remain largely unclear. Recently, tomato *SlGH3.4* was suggested to negatively regulate arbuscule incidence *via* decreasing auxin levels in colonized cells. In this study, by acquiring rice *OsGH3.2pro*:β-glucuronidase (*GUS*) transgenic plants and generating *Osgh3.2* mutants *via* CRISPR/Cas9 technique, the roles of *OsGH3.2* in modulating rice root morphology and affecting AM symbiosis were investigated through time course experiments. Unlike *SlGH3.4*, *OsGH3.2* showed asymbiotic expression in rice young lateral roots, and its mutation resulted in a “shallow” root architecture. Such root morphological change was also observed under symbiotic condition and it likely promoted AM fungal colonization, as the mutants exhibited higher colonization levels and arbuscule incidence than wild-type at early stages. Similar to *SlGH3.4*, *OsGH3.2* showed symbiotic expression in cortical cells that have formed mature arbuscules. At late stages of symbiosis, *Osgh3.2* mutants showed elongated cortical cells and larger arbuscules than wild-type, indicating elevated auxin level in the colonized cells. Together, these results revealed both asymbiotic and symbiotic roles of *OsGH3.2* in modulating rice root architecture and controlling auxin levels in arbusculated cells, which further affected colonization rate and arbuscule phenotype.

## Introduction

Arbuscular mycorrhiza (AM) is a symbiosis formed between most land plants from bryophytes to angiosperms and soil fungi of the subphylum Glomeromycotina (Wang and Qiu, 2006; Smith and Read, 2008; Spatafora et al., 2016). Its establishment involves a series of complex and well-coordinated events. Before physical contact, plant roots secrete a variety of compounds to trigger nearby AM fungi (AMF) for hyphae elongation and branching (Akiyama et al., 2005; Nagahashi and Douds, 2011), while AMF also produce chitinaceous signals to pre-activate root cells (Maillet et al., 2011; Genre et al., 2013). After touching the root surface, AMF form adhesion structures called hyphopodia, then invade the roots *via* pre-penetration apparatuses (PPA), a tunnel structure assembled by plants (Genre et al., 2005, 2008, 2012). By reaching the inner cortex, fungal hyphae spread longitudinally and penetrate inside cortical cells, developing highly branched, bush-like structures called arbuscules (Genre et al., 2008). Arbuscules are surrounded by a plant-derived plasma membrane called the peri-arbuscular membrane (PAM). Through the interface of PAM and arbuscules, nutrient exchanges occur between symbiotic partners, where plants provide organic carbon to fungi and fungi supply mineral nutrients, especially phosphate and nitrate, to plants (Smith and Smith, 2011; Wang et al., 2017, 2020; Wipf et al., 2019).

As key regulators of plant physiological and developmental processes, many phytohormones have been found to play roles in AM symbiosis (Foo et al., 2013; Gutjahr, 2014; Pozo et al., 2015). Strigolactones (SLs), a class of carotenoid-derived hormone, are secreted by plant roots to promote hyphae branching and to accelerate AMF colonization (Akiyama et al., 2005; Gomez-Roldan et al., 2008); while salicylic acid (SA), a defense-related phytohormone, shows an antagonistic delay effect on AMF penetration and colonization (Herrera-Medina et al., 2003). Auxins, abscisic acid (ABA) and jasmonic acid (JA) have been all found to positively regulate arbuscule development, while gibberellins (GA) conversely inhibit arbuscule formation (Isayenkov et al., 2005; Herrera-Medina et al., 2007; Foo et al., 2013; Etemadi et al., 2014; Pozo et al., 2015). Large knowledge gaps still remain for the detailed regulation mechanisms by phytohormones in AM symbiosis.

Only recently, genetic and molecular studies started to uncover the regulatory roles of auxin in AM symbiosis. In tomato (*Solanum lycopersicum*), examining the auxin-signaling defective mutant *diageotropia* (*dgt*) and the auxin hyper-transporting mutant *polycotyledon* (*pct*) revealed striking differences in AM phenotype (Hanlon and Coenen, 2011). Growing no lateral roots, the root organ cultures (ROCs) of *dgt* mutant showed no colonization by AMF, and the fungal hyphae even curled away from the roots. By contrast, the ROCs of *pct* grew more lateral roots and had enhanced fungal colonization with more arbuscules. In pea, an auxin-deficient mutant *bushy* (*bsh*) showed reduced colonization, accompanied by a significant decrease of SL exudation and 10 times lower expression of a SL-biosynthesis gene, *CCD8* (Foo, 2013). Moreover, a tomato gene *SlIAA27* encoding an auxin response regulator was up-regulated at the pre-contact stage of AM symbiosis, and silencing this gene resulted in down-regulation of several SL-biosynthesis genes and low AMF colonization rate (Guillotin et al., 2017). Further evidence supporting a function of auxin signaling in arbuscular development came from a study of microRNA393 (miR393), which is a negative regulator of the plant auxin receptors TIR1 (transport inhibitor response1) and AFB (auxin-related F box) (Etemadi et al., 2014). Overexpression of miR393 resulted in strong defects in arbuscule formation in three different plant species, *Medicago truncatula*, rice (*Oryza sativa*) and tomato. A Direct-repeat5 (DR5)-β-glucuronidase (GUS) promoter serving as an indicator of auxin response was also activated in the arbuscule-containing cells (Etemadi et al., 2014). In *Brachypodium distachyon*, a mutant with higher endogenous auxin content also increased AMF colonization and arbuscule abundance (Buendia et al., 2019). Together, these studies revealed a coordinate role of auxin in regulating SL synthesis, thus promoting AMF colonization, and also supported a requirement of auxin signaling responses for arbuscular development during AM symbiosis.

In plant cells, free auxin levels are determined by a balance of transport, synthesis, storage, and degradation mechanisms, and indole-3-acetic acid (IAA) is the most abundant form (Casanova-Sáez and Voß, 2019). By conjugating IAA to amino acids for storage or degradation, members of the *GRETCHEN HAGEN 3* (*GH3*) family encoding acyl acid-amido synthetases are critical for maintaining auxin homeostasis (Staswick et al., 2005). With its first member identified in soybean as an auxin rapid response gene (Hagen and Guilfoyle, 1985), *GH3* family has a total of 19 and 13 genes in *Arabidopsis thaliana* and rice genomes, respectively, and these genes were phylogenetically divided into three groups (Staswick et al., 2002; Terol et al., 2006; Okrent and Wildermuth, 2011). In *Arabidopsis*, a *GH3* group II member, *AtGH3.6* (*DFL1*, dwarf in light 1) showed a function of negatively regulating shoot and hypocotyl cell elongation as well as lateral root formation under light conditions (Nakazawa et al., 2001), and *in vitro* experiments demonstrated that AtGH3.6 can conjugate IAA to several amino acids, especially Asp (Staswick et al., 2005). Another gene *AtGH3.17* in group III also exhibited a function of inhibiting hypocotyl elongation, and mutation of *AtGH3.17* increased free IAA levels in hypocotyl at the expense of IAA-Glu (Zheng et al., 2016). Both IAA-Asp and IAA-Glu result in IAA degradation in *Arabidopsis* (Ludwig-Müller, 2011; Casanova-Sáez and Voß, 2019). In rice, two *GH3* group II genes, *OsGH3.8* and *OsGH3.2* were reported to suppress IAA-induced expressions of cell wall expansin genes, consequently enhancing plant basal immunity to pathogens (Ding et al., 2008; Fu et al., 2011). Overexpressing *OsGH3.8* resulted in several-fold higher IAA-Asp in rice leaves, indicating a function of conjugating free IAA for degradation (Ding et al., 2008). Similarly, OsGH3.2 conjugates IAA to Asp *in vitro* and overexpressing *OsGH3.2* in rice caused a dwarf phenotype associated with low IAA levels (Fu et al., 2011; Du et al., 2012).

Considering the critical roles of plant *GH3* genes in maintaining auxin homeostasis and the positive roles of auxin in AM symbiosis, especially for arbuscule development, it was wondered whether some *GH3* family members are involved in AM symbiosis. In tomato, one *GH3* gene, *SlGH3.4* was found strongly up-regulated by AMF inoculation and it was predominantly expressed in cells forming arbuscules (Liao et al., 2015). Transcriptome data for several other angiosperms including *O. sativa* (Fiorilli et al., 2015; Gutjahr et al., 2015; Roth et al., 2018), *Sorghum bicolor* (Watts-Williams et al., 2019), *M. truncatula* (Garcia et al., 2017; Luginbuehl et al., 2017), and *Populus trichocarpa* (Calabrese et al., 2017) also indicate that certain *GH3* genes in these species are induced during AM symbiosis. Little is known about the exact roles of *GH3* genes in AM symbiosis. Only recently, a study reported that tomato *SlGH3.4* could negatively regulate AM symbiosis, especially arbuscule incidence in mycorrhizal roots, likely by maintaining auxin homeostasis in colonized cortical cells (Chen et al., 2021). More aspects remain to be investigated, though, such as asymbiotic expression patterns of *GH3* genes and mutant phenotypes at different stages of symbiosis. In this study, by acquiring rice *OsGH3.2pro*:*GUS* transgenic plants and generating *Osgh3.2* mutants *via* CRISPR/Cas9 technique, the roles of *OsGH3.2* in modulating rice root morphology and also affecting AM symbiosis were revealed through time-course experiments.

## Materials and Methods

### Reconstructing a Phylogeny of Plant *GH3* Gene Family

A consensus sequence of GH3 domain (Pfam03321) was downloaded from the NCBI Conserved Domain Database^1^ and used to run BLASTP searches against the proteomes of 26 representative green plants (for species list and detailed genome information, see Supplementary Table 1). Sequences showing an *E*-value < 10^–4^ and >50% length coverage were kept and further examined on Pfam^2^ to confirm the presence of GH3 domain. All the identified plant *GH3* sequences were aligned by ClustalW and Muscle programs, which are implemented in MEGA 7.0 (Kumar et al., 2016). A phylogenetic tree of *GH3* family was then reconstructed using the aligned protein sequences by adopting a maximum-likelihood method under the JTT + I + G4 model *via* IQ-TREE, and the robustness of internal branches was tested by calculating the Shimodaira–Hasegawa approximate likelihood ratio test (SH-aLRT) and by performing 1000 ultrafast bootstrap replicates (Trifinopoulos et al., 2016).

### Plant Cultivation and the Inoculation With *Rhizophagus irregularis*

The rice (*O. sativa* ssp. japonica) wild-type and transgenic plants used in this study were in the cv. Nipponbare background. The rice seeds were surface-sterilized with 2.5% sodium hypochlorite solution for 20 min, washed extensively with sterile water and germinated on a modified 1/2 Murashige-Skoog (MS) medium. Two-week-old rice seedlings were planted in pots (12 cm in diameter, three seedlings/pot) containing a mixture of autoclaved sand and perlite (3:1, V/V). For AM colonization, about 150 spores of *R. irregularis* freshly extracted from carrot hairy root co-cultures were added to the rhizosphere of each seedling. For mock inoculation, only the washed distilled water without spores were added. Plants were placed in a growth room under a 16/8 h day/night cycle at 28°C/22°C and were fertilized twice a week with 1/2 Hoagland solution containing 20 μM phosphates. Plants were harvested at several time points, including 1-week post inoculation (wpi), 3-wpi, 5-wpi, 7-wpi, and 9-wpi. Normally, half collected root samples were used for staining/mycorrhizal phenotype examination and the other half were frozen in liquid nitrogen and kept at –80°C for RNA extraction.

For hydroponic culture, surface-sterilized rice seeds were germinated in sterile water for 2 days at 37°C, then transferred into hydroponic boxes (length 12.6 cm, width 8.5 cm, height 11 cm, 16 seedlings/box). The plants were grown in sterile water for 1 week, then in 1/4 Hoagland solution for another 2 weeks. To compare the root morphology between wild-type rice and the mutants, the root length of the longest crown root (CR), the total number of CRs, and the total number of large lateral roots (LLRs) were recorded each week. At the end of this experiment, the root samples of both wild-type and *Osgh3.2-1* mutant were sent to measure the free IAA levels commercially *via* the AB Sciex QTRAP 6500 LC-MS/MS platform (MetWare, Wuhan, China).

### RNA Extraction and Quantitative RT-PCR

In a time-course inoculation experiment, root samples of three wild-type rice individuals were collected at multiple time points and further used to examine the expression patterns of 13 *GH3* genes. For either wild-type or the *Osgh3.2* mutants, root samples of three individuals were also collected at different time points and used to evaluate the expression patterns of *OsPT11* (*phosphate transporter 11*), *OsAFB2* (*auxin-related F box 2*) and *OsD17/CCD7* (*dwarf 17*/*carotenoid cleavage dioxygenase 7*) genes. Total RNAs of rice roots were extracted by the Trizol method. The RNA purity and integrity were examined by RNA electrophoresis in 2.5% (w/v) agarose gel. One μg RNA was treated with DNase I prior to cDNA synthesis using a Revert-Aid First Strand cDNA Synthesis Kit (Thermo Scientific, Waltham, MA, United States). Quantitative RT-PCR (qRT-PCR) was performed by measuring the intensity of SYBR Green Fluorescent dye conjugated to double stranded DNA molecules using a C1000 Thermal Cycler Real-Time PCR detection system (Bio-Rad, Hercules, CA, United States). Expression values of interested genes were normalized to rice *ubiquitin 1* gene (*OsUBI1*, Chen et al., 2008), and analyzed with the 2^–Δ^ *^CT^* method (Livak and Schmittgen, 2001). Statistical differences were evaluated *via* the Student’s *t*-test. All the qRT-PCR primers used are provided in Supplementary Table 2.

### Constructing the *OsGH3.2pro:GUS* Vector and GUS Staining

A 2.0 kb fragment upstream of the *OsGH3.2* start codon was amplified from rice genomic DNA (for primers, see Supplementary Table 2) using a high fidelity *Fastpfu* enzyme and cloned into pEASY-Blunt Zero vector by TA cloning (Transgene, Beijing, China). The fragment was then digested from the vector and ligated with a pre-digested linear binary vector pBI101.1. The constructed pBI101.1 vector containing the *OsGH3.2pro:GUS* reporter unit was introduced into rice *via Agrobacterium tumefaciens*-mediated transformation, as described in Liu et al. (2021). Positively transformed seedlings were identified by PCR using the primers *OsGH3.2pro-F* and *PBI101.1-GUS-R* (Supplementary Table 2) and selected for seed propagation. The transformed T_1_ plants were then used to examine the expression activities of the *OsGH3.2* promoter in rice roots under both asymbiotic and symbiotic conditions. The rice roots were incubated at 37°C in GUS staining solution (100 mM sodium phosphate buffer, pH 7.0, 10 mM EDTA, 0.5mM K_4_[Fe(CN)_6_], 0.5 mM K_3_[Fe(CN)_6_], 0.1% Triton X-100, 20% (w/v) methanol and 0.5 mM X-Gluc) for 5 h, then fixed by FAA (5% formaldehyde, 5% ethanoic acid, and 50% ethanol) and dehydrated with ethanol. The treated roots were observed under a Nexcope-NE620 light microscope. To visualize the fungal structures, the roots were counterstained further by WGA-Alexa Fluor 488 as described below.

### CRISPR/Cas9 Vector Construction and Obtaining *Osgh3.2* Mutant Lines

To generate *Osgh3.2* mutants, the Clustered Regularly Interspaced Short Palindromic Repeats (CRISPR)-Cas9 technique was employed. Single-guide RNA (sgRNA) targeting *OsGH3.2* was designed using the CRISPRdirect^3^ online tool (Naito et al., 2015). Two best-scored sgRNAs (sgRNA1 and sgRNA2) showing the least off-target possibilities were selected, with their spacer sequences (upstream of the protospacer adjacent motif sites) matching nucleotides 2151–2170 and 2271–2290 in exon 3 of *OsGH3.2*, respectively (Supplementary Figure 3A). For each sgRNA, a pair of complementary primers with sticky ends were synthesized, annealed, and ligated with a linear CRISPR vector, pRGEB31, which was pre-digested by the restriction enzyme, *Bsa*I. The pRGEB31-sgRNA1 and pRGEB31-sgRNA2 vectors were then introduced into rice calli. The edited alleles of *OsGH3.2* were examined among transformed rice seedlings through PCR and sequencing with a pair of primers, *OsGH3.2 edit-F/R*, which cover both editing regions. In subsequent generations, plants carrying desired homozygous mutations without the pRGEB31 vector, which can be detected by *pRGEB31-Cas9-F/R* primers, were selected for reproduction. Finally, T_2_ or T_3_ generations of two mutant lines (*Osgh3.2-1*, *-2*) were used for further studies. All the primers used are provided in Supplementary Table 2.

### Fungal Structure Staining, Quantification and Microscopic Imaging

For wheat germ agglutinin (WGA) staining (Schaffer and Peterson, 1993), fresh rice roots were fixed in 50% ethanol for >4 h and cleared in 20% KOH (w/v) for 2–3 days at room temperature. Next, the roots were acidified in 0.1 M HCl for 20 min and rinsed five times with phosphate-buffered saline (PBS) solution (pH 7.4). After incubating overnight in PBS solution containing 1 μg/mL WGA-Alexa Fluor 488 (Invitrogen, Carlsbad, CA, United States) or further with 10 μg/mL Propidium iodide (Sangon Biotech, Shanghai, China), the stained roots were examined under a confocal laser scanning microscope (ZEISS LSM980) and representative images were captured. For ink-vinegar staining (Vierheilig et al., 1998), rice roots were cleared with 20% KOH for 40–120 min at 65°C, acidified by 5% acetic acid for 5 min at room temperature, incubated in ink-vinegar solution for 30 min at 65°C, and decolored in tap water for 14 h. The stained roots were examined by a Nexcope-NE620 light microscope. To quantify the total colonization rate, a modified gridline intersect method (McGonigle et al., 1990) was used to score fungal structures.

### Measuring Longitudinal Lengths of Cortical Cells and Arbuscules

After Ink-vinegar staining, the images of colonized root fragments (see an example in Supplementary Figure 7) were taken by JieDa microscopic imaging software and the longitudinal lengths of cortical cells and arbuscules were measured by using a ruler implemented in the software. Also, the total length of infection units from hyphopodia to the running edge of hyphae toward the root tip and the longitudinal length of neighboring non-colonized cortical cells were measured. In total, for either wild-type rice or the mutants, at least 15 root fragments from three individuals were measured at 3-wpi.

At 7- and 9-wpi, non-colonized cells were hard to be found since lateral roots had been heavily colonized. The longitudinal length of an array of colonized cortical cells and their contained arbuscules from individual infection site (hyphopodia) toward the root tip were measured in roots of wild-type rice and both *Osgh3.2* mutants. The arbuscules population were further sorted into three size categories, small/degenerate (<30 μm), middle (30–50 μm), and large (>50 μm) according to their longitudinal length. For each genotype, three rice individuals were included and for each rice individual, 100 colonized cells from an average of 10 infection sites were measured.

## Results

### Multiple Arbuscular Mycorrhizal-Induced *GH3* Genes Belong to the Subgroup II-A of *GH3* Gene Family

**FIGURE 1:**
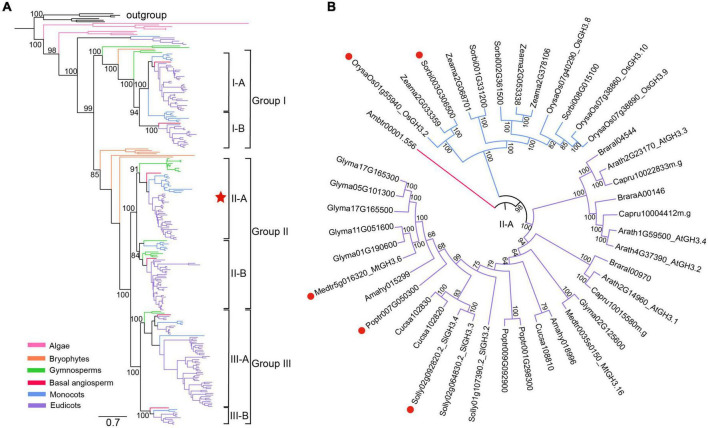
Phylogenetic analyses of plant *GH3* genes. **(A)** A reconstructed phylogeny of plant *GH3* gene family using a maximum-likelihood method under the JTT + I + G4 model *via* IQ-TREE (Trifinopoulos et al., 2016). The robustness of internal branches was evaluated by calculating the Shimodaira–Hasegawa approximate likelihood ratio test (SH-aLRT) and by performing 1000 ultrafast bootstrap replicates. Six bacterial *GH3* genes were designated as the outgroup. A total of 250 *GH3* genes (22 plant species) identified from algae, bryophytes, gymnosperms, basal angiosperm, monocots and eudicots were covered (for a complete tree showing all sequence names, please refer to Supplementary Figure 1). The angiosperm *GH3* genes were classified into three major groups and six subgroups, with supporting values of ultrafast bootstrap test shown on branches of these clades. The bar represents number of nucleotide substitutions per site. **(B)** A circled phylogeny of subgroup II-A (labeled by a red star in (a) covering surveyed angiosperm species. The AM-induced *GH3* genes from five angiosperms, as supported by transcriptome data, were indicated by red dots.

To first explore the evolutionary history of *GH3* gene family in plants, a total of 250 *GH3* genes (Supplementary Table 1) were identified from 22 surveyed plant species, including 215 genes identified from 13 angiosperms. Based on their protein sequences, a phylogeny of *GH3* family was then reconstructed (Figure 1A and Supplementary Figure 1). On the phylogeny, the algal *GH3* sequences occupied the most basal position (Figure 1A and Supplementary Figure 1), suggesting that an ancestral *GH3* gene had originated in the algal ancestor. This ancestral *GH3* gene further divided into two separate lineages in bryophytes, as represented by two *P. patens GH3* genes, *PpGH3.1* (*Pp3c24_16260*) and *PpGH3.2* (*Pp3c10_20960*), which share 43.4% identities at the protein level (Figure 1A and Supplementary Figure 1). More *GH3* lineages further evolved in seed plants. To be largely in accordance with previous classification system covering *Arabidopsis* and rice *GH3* genes (Staswick et al., 2002; Terol et al., 2006; Okrent and Wildermuth, 2011), three major angiosperm lineages (groups I, II, III) were distinguished, with each further separated into two sublineages (subgroups I-A, I-B, II-A, II-B, III-A and III-B) (Figure 1A and Supplementary Figure 1). It was reported that a tomato *GH3* gene, *SlGH3.4*, is strongly induced in mycorrhizal roots (Liao et al., 2015). Based on the reconstructed phylogeny, *SlGH3.4* belongs to the subgroup II-A (Figure 1B). Also, transcriptome data of AM symbioses are available for some other angiosperm species. Therefore, additional AM-induced *GH3* genes were identified, including *OsGH3.2* in rice, *Sorbi003G306500* in *S. bicolor*, *MtGH3.6* in *M. truncatula*, and *Poptr007G050300 in P. trichocarpa*. Interestingly, all these genes belong to the subgroup II-A (Figure 1B), suggesting that these genes may have similar function during AM symbiosis. To explore such function, in this study, we focused on the rice *OsGH3.2* gene for further investigations.

### *OsGH3.2* Showed Asymbiotic Expression in Rice Young Lateral Roots and Symbiotic Expression in Cortical Cells That Have Formed Mature Arbuscules

**FIGURE 2:**
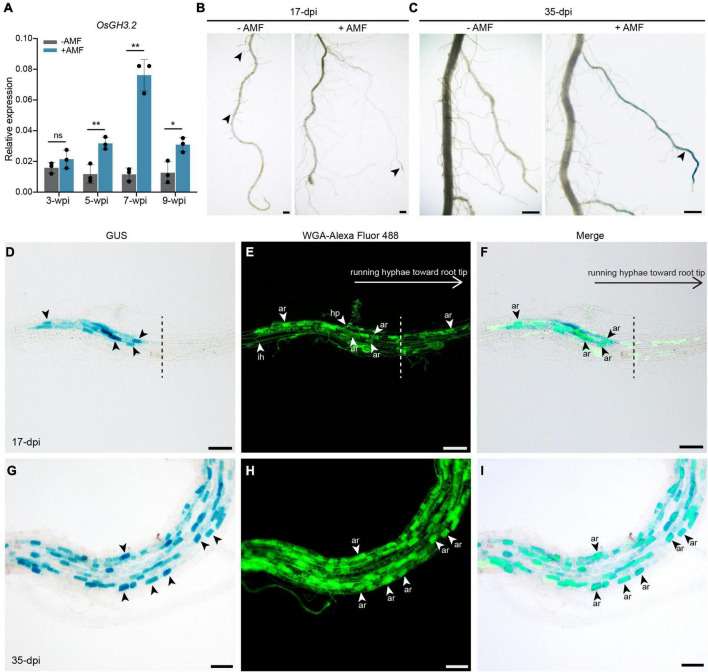
Promoter activity of *OsGH3.2* in mock-inoculated and *R. irregularis*-inoculated rice roots. **(A)** The relative expression levels of *OsGH3.2* in wild-type rice roots were measured by qRT-PCR at 3-, 5-, 7-, and 9-wpi by *R. irregularis*. A housekeeping gene, *OsUBI1*, was used for normalization. Data represent mean ± standard deviations (SD), *n* = 3. Statistical analysis was performed with Student’s *t*-test (ns, not significant; *p ≤ 0.05; **P ≤ 0.01); **(B)** A representative crown root with GUS activity detected in many young lateral roots of mock inoculated (–AMF) roots and in a large lateral root of *R. irregularis* inoculated (+AMF) roots at 17-dpi; **(C)** after 35-days inoculation, a representative mock inoculated crown root showed no more GUS activity in mature LLR, while the GUS activity were shown in more mature LLRs of *R. irregularis* inoculated roots. The roots showed GUS activity were then counterstained with WGA-Alexa Fluor 488 to visualize fungal structures at 17- and 35-days post inoculation (dpi) by *R. irregularis*. **(D–F)** GUS activities were detected in cortical cells containing mature arbuscules below or near the hyphopodia; at the side of running hyphae toward root tip (separate with a dashed line), no GUS activities were detected in colonized cells. **(G–I)** After 35-dpi, GUS activities were detected in more mature arbuscules. Arrowheads indicate various of fungal structures: hp, hyphopodia; ih, intraradical hyphae; ar, arbuscules. The scale bars represent 1 mm **(B,C)** and 100 μm **(D–I)**.

The RT-PCR results showed that seven out of 13 *OsGH3* genes appear to be expressed in rice roots under both asymbiotic and symbiotic conditions (Supplementary Figure 2A). Quantitative RT-PCR (qRT-PCR) experiments were then performed for the seven *GH3* genes. As Figure 2A showed, at 3-weeks post inoculation (wpi) by AM fungus *Rhizophagus irregularis*, *OsGH3.2* expression level was only slightly higher in mycorrhizal roots than in control roots (1.3-fold, *p* = 0.13). At 5-wpi, however, *OsGH3.2* showed a 2.7-fold induction in mycorrhizal roots (*p* < 0.01), thus confirming previous transcriptome data. As for the other six *GH3* genes, their expression patterns were not altered at a statistically significant level at either 3- or 5-wpi by *R. irregularis* (Supplementary Figure 2B). The expression level of *OsGH3.2* in mycorrhizal roots further increased at 7-wpi (6.6-fold to control, *p* < 0.01), then decreased at 9-wpi (2.4-fold to control, *p* < 0.05, Figure 2A). Such pattern seemed to be consistent with the arbuscule abundance level in mycorrhizal roots, as many arbuscules would become degenerated at late stage of symbiosis.

To visualize the spatial expression pattern of *OsGH3.2*, an *OsGH3.2pro:GUS* vector was constructed and transformed into rice to obtain stable transgenic lines (see section “Materials and Methods”). The transgenic T_1_ rice plants were either inoculated with *R. irregularis* or with mock solution and the GUS activities were examined in roots at two time points. At 17-days post inoculation (dpi) by mock solution, blue stains were seen in rice young lateral roots (Figure 2B). Examination at a later time point (35-dpi) then showed no more asymbiotic expression of *OsGH3.2* in rice mature large lateral roots (LLRs, Figure 2C). Differently, at 17- and 35-dpi by *R. irregularis*, GUS activity was mainly detected in rice mature LLRs (Figures 2B,C). To know whether fungal structures are associated with symbiotic expression of *OsGH3.2*, we then counterstained the mycorrhizal roots with WGA-Alexa Fluor 488. At 17-dpi, some fragments of LLR showed individual infection unit by AMF, and GUS activity was mainly detected in the cortical cells below or near the hyphopodia, which had formed mature arbuscules (Figures 2D–F). At the side of running hyphae toward root tip, however, no GUS activity was detected despite some cortical cells had formed mature arbuscules (Figures 2D–F). Such observation made at early symbiotic stage thus indicate that symbiotic induction of *OsGH3.2* in cortical cells is not fully synchronical to arbuscule maturation. At 35-dpi, as a large fraction of LLRs had been colonized, GUS activity were detected in many more cortical cells that had formed mature arbuscules (Figures 2G–I).

### CRISPR/Cas9-Mediated Mutagenesis of *OsGH3.2* Gene

To explore the roles of *OsGH3.2* in rice root development and in AM symbiosis, we tried to obtain null *Osgh3.2* mutants *via* CRISPR/Cas9 technique. Regenerated rice seedlings were screened for edited *Osgh3.2* alleles. A total of six mutated alleles were detected in the T_0_ generation and two of these, *Osgh3.2-1* and *Osgh3.2-2*, were selected to reach homozygosity in subsequent generations, meanwhile eliminating the pRGEB31 vector (Supplementary Figure 3). By introducing a 2-nucleotide deletion, *Osgh3.2-1* allele had altered reading frame, and consequently encoded a protein sharing no homology with the normal protein beyond amino acid site 299. Also, by introducing a 4-nucleotide deletion, *Osgh3.2-2* allele had a premature stop codon and thus encoded a truncated protein only 298 aa in length. Therefore, for both mutated alleles, their encoded proteins only retain the N-terminal half of the GH3 domain (Supplementary Figure 3C). Since the proper function of GH3 enzymes requires both the N-terminal domain and the C-terminal domain (Westfall et al., 2012), it can be expected that the mutated *Osgh3.2-1* and *-2* alleles probably lost the function of *OsGH3.2*.

### *OsGH3.2* Has Developmental Roles in Modulating Rice Root Architecture, Panicle Traits, as Well as Grain Length

**FIGURE 3:**
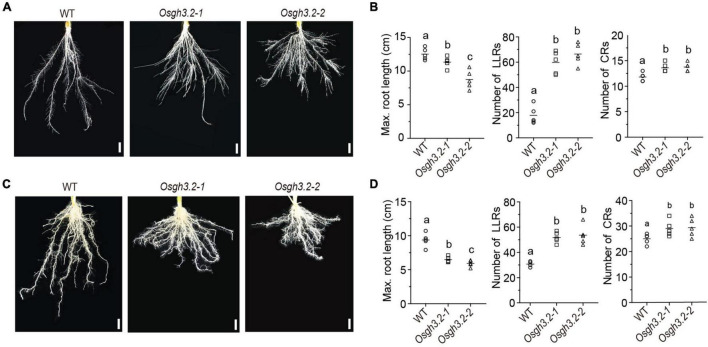
*Osgh3.2* mutants showed an altered “shallow” root architecture. Rice roots of wild-type, *Osgh3.2-1* and *Osgh3.2-2* after hydroponic culture **(A)** or inoculation by *R. irregularis* **(C)** for 3 weeks. Scale bars, 1 cm. **(B,D)** Maximum length of crown roots (CRs), number of large lateral roots (LLRs) and number of CRs were compared between wild type rice and the *Osgh3.2* mutants under two cultivation conditions, respectively. Data are individual values from five biological replicates and the horizontal line shows the mean value. Statistical analysis was performed with Student’s *t*-test. Different letters indicate statistically significant differences (*P* < 0.05).

Under asymbiotic condition, the expression of *OsGH3.2* in rice young lateral roots prompted us to explore its potential role in regulating rice root development. Seeds of wild-type rice and the *Osgh3.2* mutants were germinated and cultured hydroponically (see section “Materials and Methods”). After 3 weeks, both *Osgh3.2* mutants exhibited a “shallow” root architecture compared to wild-type (Figure 3A). The mutants had shorter maximum crown root (CR) length and grew more LLRs (∼3.4 times) and CRs (∼1.2 times) than wild-type (Figure 3B). To explore whether such phenotypic changes in mutant roots are related to altered auxin levels, free IAA levels were measured for root samples of wild-type rice and *Osgh3.2-1*. The mutant had an average of 4.1 ng IAA/g fresh root, which is indeed higher than that in wild-type rice (3.2 ng IAA/g fresh root, *p* < 0.05, Supplementary Figure 5A). Meanwhile, tryptophan, the IAA synthetic precursor, also accumulated more in *Osgh3.2-1* roots (Supplementary Figure 5B).

We further investigated whether the alteration of root architecture can still be observed under symbiotic condition. As shown in Figures 3C,D, similar morphological changes also occurred in *Osgh3.2* mutants after 3-weeks inoculation by *R. irregularis*, demonstrating that the developmental role of *OsGH3.2* in modulating rice root architecture is independent of AMF inoculation. At intermediate time points (1- or 2-week), the root morphological changes could be also detected in *Osgh3.2* mutants under either asymbiotic or symbiotic condition (Supplementary Figures 4A–D).

To see whether *OsGH3.2* has a regulatory role in above-ground tissues, we also examined the panicle traits and grain sizes of wild-type rice and the *Osgh3.2* mutants (Supplementary Figures 6A,B). Both mutants showed longer panicle length, more primary panicle branches, and *Osgh3.2-2* also showed more secondary panicle branches than wild-type rice (Supplementary Figure 6C). As for grains, the mutants exhibited longer grain length, but showed no differences to wild-type rice in terms of grain width and thickness (Supplementary Figure 6D).

### Under Symbiotic Condition, the *Osgh3.2* Mutants Exhibited Higher Colonization Rates and Arbuscule Abundance Levels Than Wild-Type at Most Examined Time Points Except at 7-wpi

**FIGURE 4:**
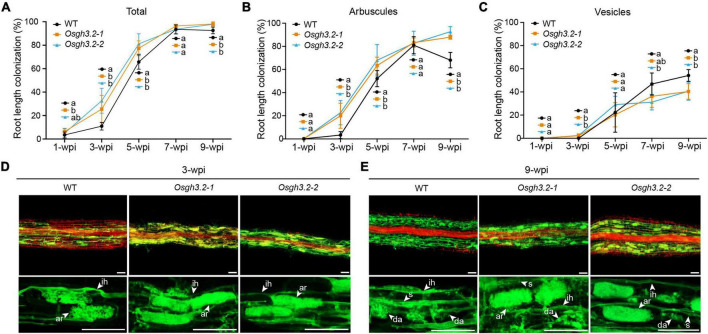
The mycorrhizal phenotypes of *Osgh3.2* mutants. **(A–C)** For both wild-type rice and the *Osgh3.2* mutants, the total root length colonization rates (%) as well as the rates of specific fungal structures including arbuscules, and vesicles were determined by the gridline intersect method at 1-, 3-, 5-, 7-, and 9-wpi by *R. irregularis.* Data represent mean ± standard deviations (SD), *n* = 4∼6. Different letters indicate statistically significant differences (Student’s *t*-test, *P* < 0.05). **(D,E)** Laser scanning confocal images showing fungal structures stained by WGA-Alexa Fluor 488 (green) and plant cell walls stained by propidium iodide (red) in wild-type rice and the *Osgh3.2* mutants at 3- and 9-wpi, respectively; ih, intraradical hyphae; ar, arbuscules; da, degenerating arbuscules; s, septa. scale bar = 50 μm.

To explore how *OsGH3.2* expressions would affect AM symbiosis, the mycorrhizal phenotypes of the two *Osgh3.2* mutants were compared to wild-type in a time course experiment. After inoculation by *R. irregularis* for 1 week, both hyphopodia and intraradical fungal hyphae were observed in rice LLRs, with *Osgh3.2-1* exhibiting a higher colonization rate than wild-type rice (6.4% vs. 3.5%, *p* < 0.05, Figure 4A). Nearly no arbuscules or vesicles were formed at this early stage (Figures 4B,C), so the colonization rate difference is unlikely to be attributed to symbiotic expression of *OsGH3.2* in arbusculated cells. At 3-wpi, the colonization rate in wild-type rice LLRs reached ∼11%, with arbuscules seen in 3% root length and still no vesicle formed. Strikingly, both *Osgh3.2* mutants showed > 25% colonization by AMF, with 20% and 2% root length forming arbuscules and vesicles, respectively (Figures 4A–C). The morphology of arbuscules formed in mutant roots was as normal as in wild-type, suggesting loss function of *OsGH3.2* would not impair arbuscular development (Figure 4D). At 5-wpi, the total colonization rate in wild-type rice LLRs further increased to 66%, while in both mutants reached a higher rate of ∼77% (Figure 4A). The arbuscule abundance level was also higher in mutant roots, though comparable numbers of vesicles were observed between wild-type and the mutants (Figures 4B,C). Together, these results illustrated that the *Osgh3.2* mutants were colonized by *R. irregularis* at higher rates than wild-type rice from 1- to 5-wpi.

The mycorrhizal phenotypes were observed continuously. At 7-wpi, the total colonization rate by *R. irregularis* in both wild-type rice and the *Osgh3.2* mutants had reached > 90% and entered a plateau phase (Figure 4A). At this time point, the mutants showed similar arbuscular abundance levels to wild-type rice (Figure 4B), though less lipid-storage vesicles were observed in the *Osgh3.2-2* mutant (Figure 4C). At 9-wpi, a majority of arbuscules observed in wild-type rice LLRs became degenerated (Figure 4E), so the arbuscule abundance level declined to 68% and the vesicle abundance level increased to 54% (Figures 4B,C). For both *Osgh3.2* mutants, however, the arbuscular abundances were maintained at even higher levels than at 7-wpi, with many arbuscules still occupying the cortical cells rather fully (Figures 4B,E). Also, the vesicle abundance level in mutant roots was much lower than in wild-type (Figure 4C).

### At Late Stage of Symbiosis, the *Osgh3.2* Mutants Showed Elongated Colonized Cells as Well as Larger Arbuscules Than Wild-Type

**FIGURE 5:**
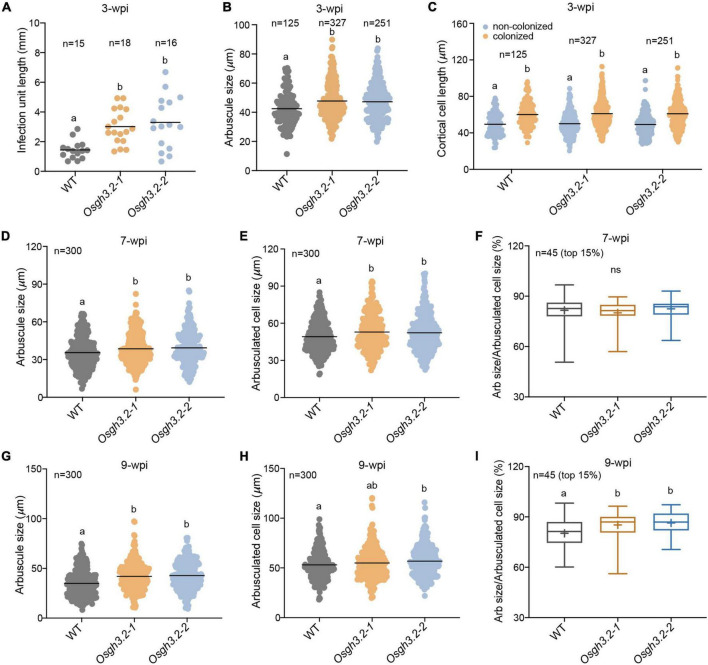
*Osgh3.2* mutants showed larger arbuscules as well as enlarged arbuscule-containing cortical cells at 7- and 9-wpi. **(A)** Lengths of infection units in wild-type and the *Osgh3.2* mutants at 3-wpi by *R. irregularis.* Data are individual values from at least 15 individual infection units from three biological replicate samples of wild-type and *Osgh3.2* mutants. **(B)** Data represent arbuscule sizes (longitudinal length) of wild-type (125 arbuscules), *Osgh3.2-1* (327 arbuscules), and *Osgh3.2-2* (251 arbuscules) at 3-wpi from all the measured infection units in **(A)**. **(C)** The measured longitudinal lengths of colonized and neighboring non-colonized cortical cells in wild-type (125 cells each), *Osgh3.2-1* (327 cells each), and *Osgh3.2-2* (251 cells each) at 3-wpi. **(D,G)** For either wild-type rice or the *Osgh3.2* mutants, the longitudinal lengths of 300 arbuscules from three rice individuals were measured. **(E,H)** The longitudinal lengths of cortical cells containing arbuscules in wild-type and the *Osgh3.2* mutants at 7- and 9-wpi, respectively. Data represent individual values of 300 colonized cells from three biological replicate samples of wild-type and *Osgh3.2* mutants. **(A–E,G,H)** The horizontal line shows the mean value. Statistical analysis was performed with Student’s *t*-test. Different letters indicate statistically significant differences (*P* < 0.05). **(F,I)** When only the 15% largest arbuscules were considered, the longitudinal length ratios of arbuscules to cortical cells were shown in box plots and compared between wild-type and the mutants. The boxes represent the variation between the first and third quartiles; the “+” indicates mean value, and the horizontal line indicate the median. Statistical analysis was performed with Student’s *t*-test (ns, not significant; different letters indicate statistically significant differences, *P* < 0.05).

To investigate whether *OsGH3.2* can affect root cortical cell morphology, multiple root fragments containing individual infection units (*n* = 15–18) were firstly analyzed at 3-wpi (see section “Materials and Methods”). On average, the length of infection units in the mutant roots was twice longer than in wild-type roots (Figure 5A), reflecting a more efficient colonization by AMF in the mutants. Also, larger arbuscule mean size was observed in the mutants (Figure 5B). The longitudinal lengths of colonized cortical cells and their neighboring non-colonized cells were further measured. On average, for either wild-type or the mutants, the colonized cortical cells showed longer lengths than their neighboring non-colonized cells (Figure 5C), suggesting that fungal invasion and forming arbuscules would enlarge the infected cortical cells. However, between wild-type and the mutants, neither colonized nor non-colonized cortical cells showed mean length differences (Figure 5C), suggesting that loss function of *OsGH3.2* had not caused obvious effect on the cortical cell length at this early symbiotic stage. This is consistent with the low induction level of *OsGH3.2* observed at this time point (Figure 2A).

At 7-wpi, when *OsGH3.2* had been extensively expressed in mycorrhizal roots (Figure 2A), we further measured the longitudinal lengths of colonized cortical cells from each genotype (see section “Materials and Methods”). It was found that on average, both *Osgh3.2* mutants had elongated colonized cortical cells than wild-type (Figure 5E). Measuring the lengths of arbuscules within these cortical cells also revealed larger arbuscules in the mutants (Figure 5D). When only a subset of large arbuscules (top 15%) was considered, the relative ratio of arbuscule length to cell length then showed no difference between wild-type and the mutants (81.6% vs. 81.3% on average, Figure 5F), suggesting that the larger arbuscules observed in the mutants were most likely due to extra room provided in the enlarged cortical cells. At 9-wpi, the measurements were repeated again. Since many arbuscules had started degenerating in wild-type (Figure 4E), the mutants not only exhibited longer lengths of colonized cortical cells and arbuscules (Figures 5G,H), but also showed higher relative ratio of arbuscule to cell lengths (Figure 5I), indicating that the arbuscules in the mutants were less degenerated than in wild-type at this time point. We also sorted the arbuscule population into three size categories, small/degenerate (<30 μm), middle (30–50 μm), and large (>50 μm). At 9 wpi, both mutants had maintained significantly higher ratios of large arbuscules but lower rations of small/degenerate arbuscules than wild-type (Supplementary Figure 8B), indicating that the arbuscules are less degenerated in the mutants. While at 7 wpi, the ratio differences were not statistically significant for either large or middle size arbuscules (Supplementary Figure 8A). Even for small size arbuscules, only the *Osgh3.2-2* but not *Osgh3.2-1* mutant showed significant lower ratio than wild-type (Supplementary Figure 8A), likely because the arbuscules had not entered into degeneration stage largely at 7 wpi in wild-type.

### Expression of Several Marker Genes at Different Symbiotic Stages

**FIGURE 6:**
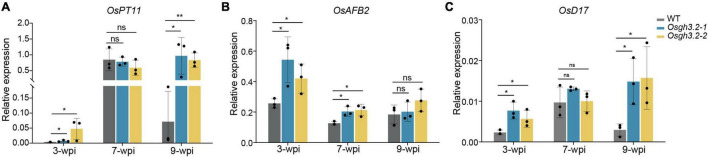
The expression patterns of *OsPT11*, *OsAFB2*, and *OsD17* at 3-, 7- and 9-wpi by *R. irregularis* in roots of wild-type rice and the *Osgh3.2* mutants. **(A–C)** Relative expression levels of *OsPT11*, *OsAFB2*, and *OsD17* were measured by qRT-PCR experiments. A housekeeping gene, *OsUBI1*, was used for normalization. Data represent mean ± standard deviations (SD) of three biological replicates. Statistical analysis was performed with Student’s *t*-test (ns, not significant; *P ≤ 0.05; **P ≤ 0.01).

Finally, the relative expression levels of several rice genes were examined at different colonization stages between wild-type and the *Osgh3.2* mutants, including *OsPT11*, an AM marker gene associated with arbuscular phosphate transferring, *OsAFB2*, an auxin response gene and *OsD17*, a gene involved in SL synthesis. The relative expression levels of *OsPT11* were in good accordance with the arbuscule abundance levels (Figure 6A), thus supporting the observed arbuscular phenotype at different time points (Figure 4B). *OsAFB2* in both mutants were expressed at significantly higher levels than in wild-type rice at 3-wpi and 7-wpi (less evident at 9-wpi), indicating more active auxin responses in the mutant roots (Figure 6B). The expression of *OsD17* gene is relevant to the SL synthesis, and stronger expression of this gene in the mutants at 3-wpi (Figure 6C) suggested a possibility of synthesizing and secreting more SLs to attract AMF colonization. Also, *OsD17* was expressed at comparable levels between wild-type and the mutants at 7-wpi, but at lower level in the wild-type at 9-wpi, reflecting a correlation with arbuscular abundance level like *OsPT11* (Figure 6C).

## Discussion

The plant *GH3* gene family contains members encoding acyl acid-amido synthetases that can modulate phytohormone levels by conjugating IAA, JA, or SA to amino acids (Staswick et al., 2005). To explore a full evolutionary history of this gene family, we identified a total of 250 *GH3* genes from 22 green plant species and further reconstructed a phylogeny (Figure 1A and Supplementary Figure 1). It turned out that an ancestral *GH3* gene had originated in the algal ancestors of land plants, and it further diverged into two lineages in bryophytes, as represented by two *GH3* genes in the moss *P. patens*. Enzyme activity assays had revealed that both PpGH3.1 and PpGH3.2 can conjugate IAA and knock-out either *PpGH3* gene increased plant sensitivity to auxin, with the mutants accumulating higher levels of free IAAs than wild type plants (Ludwig-Müller et al., 2009).

Among the 13 *GH3* genes in rice genome, seven appeared to be expressed in roots (Supplementary Figure 2A), and qRT-PCR results not only confirmed previous transcriptome data that *OsGH3.2* is induced during AM symbiosis (Fiorilli et al., 2015; Gutjahr et al., 2015; Roth et al., 2018), but further revealed that *OsGH3.2* expression level is correlated with arbuscule abundance levels at different symbiotic stages (Figures 2A, 4B). In root fragments containing individual infection units at 17-dpi, *OsGH3.2* promoter activity was mainly detected in colonized cortical cells that had formed mature arbuscules below or near to the hyphopodia (Figures 2D–F). It is interesting to observe no *OsGH3.2* promoter activity at the foreside of running hyphae toward root tips, although some cortical cells had formed mature arbuscules (Figures 2D–F). Such observation indicated that the *OsGH3.2* expression is not synchronical to arbuscule maturation, but is after mature arbuscules have been developed. It could therefore explain the low expression level of *OsGH3.2* at 3-wpi (Figure 2A), at which time mature arbuscules were not extensively formed in wild-type rice roots (Figure 4B). Similar expression pattern was also revealed for *MtGH3.6* in *Medicago.* According to the transcriptome data reported recently, *MtGH3.6* showed no obvious induction at 8-dpi but was induced 64-fold at 23-dpi during AM symbiosis (Luginbuehl et al., 2017). It is possible that these *GH3* genes are quickly induced after the free auxin level in the arbusculated cells reaches certain threshold. Previously, both *SlGH3.4* and *OsGH3.2* were revealed to be rapidly induced by exogenous IAA treatment (Fu et al., 2011; Liao et al., 2015).

In plants, auxin is a major regulator of root development, especially for lateral root formation (Du and Scheres, 2018). In *Arabidopsis*, *AtGH3.6/DFL1* negatively regulates lateral root formation (Nakazawa et al., 2001), while *AtGH3.2* mutant (*ydk1-D*) had a short primary root with a reduced number of lateral roots (Takase et al., 2004). In rice, overexpressing *OsGH3.8* displayed shorter and fewer adventitious roots (Ding et al., 2008) and overexpression of *OsGH3.2* resulted in fewer but longer crown roots (Du et al., 2012). In this study, two *Osgh3.2* mutant lines were generated *via* CRISPR/Cas9-mediated mutagenesis (Supplementary Figure 3) and the developmental roles of *OsGH3.2* were explored since this gene showed asymbiotic expression in rice young lateral roots (Figure 2B). After 3-weeks hydroponic experiment, both *Osgh3.2* mutants showed altered root architecture, with more CRs and LLRs developed, while the maximum length of CR was inhibited (Figures 3A,B). Increased levels of free IAA and its precursor were further detected in roots of *Osgh3.2-1* mutant (Supplementary Figure 5), supporting that the phenotypic changes are related to auxin activities. It is also in contrast with the phenotypes observed in *OsGH3.2* over-expression lines, which had reduced IAA levels (Du et al., 2012). Interestingly, no remarkable effect on plant growth and root development were observed in tomato *slgh3.4* mutants, and based on qRT-PCR data, the gene was neither expressed in roots nor in above-ground tissues under non-mycorrhizal condition (Chen et al., 2021). However, a duplicated gene *SlGH3.2* showed expression in tomato roots (Liao et al., 2015). It is possible that gene duplications had resulted in subfunctionalization in tomato.

After inoculation by *R. irregularis*, the altered root morphology was still observed in the *Osgh3.2* mutants (Figures 3C,D), indicating that the architectural change is independent of AMF colonization. Similar alterations were also observed for the panicles. On reaching maturity, the mutant panicles exhibited more primary branches and secondary branches, with the grain length also increased (Supplementary Figure 6).

We conducted a time-course inoculation experiment to investigate whether AM symbiosis would be affected in the *Osgh3.2* mutants. It was revealed that both mutants exhibited higher colonization levels than wild-type from 1- to 5-wpi by *R. irregularis* (Figures 4A–C). The altered root morphology in *Osgh3.2* mutants likely provided more potential colonization sites for fungal hyphae, thus promoted AMF colonization. Previously in tomato, the auxin hyper-transporting mutant *pct* also showed more lateral roots and enhanced fungal colonization (Hanlon and Coenen, 2011). Additionally, examining *OsD17/CCD7* expressions at 3-wpi revealed that both *Osgh3.2* mutants expressed this SL-biosynthesis gene several-fold higher than wild-type rice (Figure 6C). The mutants could therefore have more SLs to attract AMF and establish colonization more successfully. Interestingly, like *OsPT11*, *OsD17* expression levels in both wild-type and *Osgh3.2* mutants exhibited a good correlation with arbuscular abundance levels at later time points, indicating a potential role of *OsD17* and SLs in arbuscule development. Previous study by Floss et al. (2008) showed that in *Medicago*, gene *DXS2* [located at upstream of methylerythritol phosphate (MEP) pathway and SL biosynthesis] is mainly expressed in the arbusculated cells, suggesting that the arbuscule development would potentially need SLs. Further study would be required to explore whether *OsD17* is also expressed in the arbusculated cells. Also, in pea, an auxin-deficient mutant *bsh* expressed another SL-biosynthesis gene, *CCD8* at much lower level than wild-type and showed reduced colonization by AMF (Foo, 2013). It remains uncertain whether the altered root architecture, or the changed expression of SL-biosynthesis genes (or even both) leaded to the elevated colonization rate by AMF in the *Osgh3.2* mutants, but it is unlikely caused by the symbiotic expression of *OsGH3.2* in the colonized cortical cells, at least at early symbiotic stages (1- and 3-wpi) when *OsGH3.2* had not been extensively expressed in mycorrhizal roots (Figure 2A). Such results would provide new thoughts on the tomato *Slgh3.4* phenotype, which showed higher arbuscule incidence but comparable total colonization rates to wild-type at 5-wpi by AMF (Chen et al., 2021). Examining the symbiosis at earlier time points would help to elucidate when the arbuscule incidence started to increase in the *Slgh3.4* mutants.

It has been demonstrated that arbuscular development in cortical cells requires auxin signaling responses (Etemadi et al., 2014). In tomato, over-expressing *SlGH3.4* also resulted in underdeveloped arbuscules, likely due to insufficient cellular auxin levels (Chen et al., 2021). It is so far unclear on the fluctuations of free auxin levels in colonized cortical cells at different symbiotic stages. However, based on the demonstrated roles of *OsGH3.2* in conjugating IAA and reducing free IAA levels (Fu et al., 2011) and the induction pattern of *OsGH3.2* in cortical cells (Figure 2), we assume that the auxin levels in the colonized cortical cells would be down-regulated by *OsGH3.2* to maintain a cellular auxin level homeostasis after mature arbuscule have developed. This assumed function explains why the expression level of *OsGH3.2* is highly correlated with arbuscule abundance levels, which both peaked at 7-wpi (Figures 2A, 4B). At 9-wpi, when the arbuscules largely entered into degeneration phase in wild-type rice roots, the expression level of *OsGH3.2* also decreased (Figures 2A, 4B). Loss function of *OsGH3.2*, although did not impair arbuscule development, however, seemed to affect (or delay) the degeneration phase, as arbuscule abundance level further increased in the *Osgh3.2* mutants at 9-wpi, with fewer degenerating arbuscules and vesicles observed than wild-type (Figures 4B,C,E). Most likely, the *Osgh3.2* mutants were not able to down-regulate auxin levels in the colonized cortical cells to the extend as in wild-type. However, it remains unclear why plants need to induce the *GH3* genes to control the cellular auxin levels. One possibility is that elevated auxin levels may bring plants potential “hurt or danger,” e.g., pathogen invasion. Studies on *OsGH3.8* and *OsGH3.2* genes had revealed functions of suppressing IAA-induced expressions of cell wall expansin genes, consequently enhancing plant basal immunity to pathogens (Ding et al., 2008; Fu et al., 2011). Another possibility is that a lower auxin level would be advantageous to arbuscule degeneration, since previous studies have revealed that auxin signaling responses are required for arbuscule development in rice (Etemadi et al., 2014). Therefore, it is logical to deduce that the reduced IAA levels in the arbuscule-containing cells would hinder further arbuscule development and accelerate their degeneration.

A previous study in cucumber had revealed that the cortical cells containing the AM fungus were significantly larger than the corresponding cortical cells from non-mycorrhizal roots (Balestrini et al., 2005). Considering the role of auxin in cell expansion (Du et al., 2020), and *OsGH3.2* likely has a symbiotic function of down-regulating auxin levels in colonized cortical cells, we wonder whether this gene, when mutated, would affect cell sizes as well as arbuscule sizes. At 3-wpi, we examined root fragments containing individual infection units and measured both colonized and neighboring non-colonized cortical cells (Figure 5C). Although a longer longitudinal length of colonized cells than non-colonized cells was detected in each genotype, there were no statistically significant differences for the mean longitudinal lengths of either colonized or non-colonized cortical cells between wild-type and the two mutants (Figure 5C). This is consistent with the rather low expression of *OsGH3.2* among colonized cells at this early stage (Figure 2A). At 7- and 9-wpi, however, both *Osgh3.2* mutants had increased cortical cell lengths (Figures 5E,H) and larger arbuscules (Figures 5D,G) than wild-type, likely because the mutants had no functional OsGH3.2 proteins to inhibit the auxin effects in the colonized cells. When only 15% of the largest arbuscules were considered, the relative ratio of arbuscule length to their cortical cell length showed no differences between wild-type and the mutants at 7-wpi (Figure 5F), therefore suggesting that arbuscular size change observed in the mutants is most likely an indirect consequence of enlarged cortical cell sizes. At 9-wpi, as more arbuscules were degenerating in wild-type, the size ratio of arbuscule to cortical cell also showed differences between wild-type and the mutants (Figure 5I). Taken together, these data suggested that AM symbiosis can induce the expression of *OsGH3.2* in cortical cells that have formed mature arbuscules and further affect the longitudinal length of colonized cells by down-regulating cellular auxin levels. Previously, another study in *M. truncatula* had also revealed that AMF are able to modulate root cortical cell development by activating a GRAS-domain transcription factor, MIG1, which can determine both the longitudinal and radical sizes of cortical cells (Heck et al., 2016).

In summary, by paying attention to the plant *GH3* gene family, this study revealed that *OsGH3.2*, a *GH3* subgroup II-A member, can not only modulate rice root architecture and further affect colonization levels by AMF, but also can modulate colonized cortical cell longitudinal lengths and consequently affect arbuscule sizes.

## Data Availability Statement

The original contributions presented in the study are included in the article/Supplementary Material, further inquiries can be directed to the corresponding author/s.

## Author Contributions

BW and C-CL planned and designed the research, wrote the manuscript. C-CL and Y-NL performed most of the experiments, analyzed the data, and contributed equally to this work. J-FC helped in quantifying colonization rates and measuring cell/arbuscule lengths. RG and LT helped in conducting fieldwork and analyzing data. All authors contributed to the article and approved the submitted version.

## Conflict of Interest

The authors declare that the research was conducted in the absence of any commercial or financial relationships that could be construed as a potential conflict of interest.

## Publisher’s Note

All claims expressed in this article are solely those of the authors and do not necessarily represent those of their affiliated organizations, or those of the publisher, the editors and the reviewers. Any product that may be evaluated in this article, or claim that may be made by its manufacturer, is not guaranteed or endorsed by the publisher.

## Footnotes

1
https://www.ncbi.nlm.nih.gov/cdd

2
http://pfam.xfam.org/

3
http://crispr.dbcls.jp

## Supplementary Material

Supplementary Figure 1 A phylogenetic tree of plant *GH3* family containing 250 genes identified from 22 plant species. The phylogeny was reconstructed using a maximum-likelihood method under the JTT + I + G4 model *via* IQ-TREE (Trifinopoulos et al., 2016). The robustness of internal branches was evaluated by calculating the Shimodaira–Hasegawa approximate likelihood ratio test (SH-aLRT) and by performing 1000 ultrafast bootstrap replicates. Six bacterial *GH3* genes were designated as the outgroup. The bar represents number of nucleotide substitutions per site.

Supplementary Figure 2 The expression patterns of 13 *OsGH3* genes in rice roots during AM symbiosis. The transcript abundances of 13 *OsGH3* genes in rice roots at 3- and 5-wpi either by mock solution (–) or by *R. irregularis* (+) were measured by RT-PCR first. **(A)** Apart from *OsGH3.2*, six *OsGH3* genes appear to be expressed in rice roots, including *OsGH3.3*, *OsGH3.5*, *OsGH3.7*, *OsGH3.8*, *OsGH3.11*, and *OsGH3.13*. **(B)** Relative expression levels of the six *OsGH3* genes were further measured by quantitative RT-PCR. The gene expression values were normalized to the rice housekeeping gene *ubiquitin 1* (*OsUBI1*). Data represent mean ± standard deviations (SD) of three biological replicates. Statistical analysis was performed with Student’s *t*-test (ns, not significant).

Supplementary Figure 3 CRISPR/Cas9-mediated *OsGH3.2* gene mutagenesis in rice. **(A)** The *OsGH3.2* gene has three exons, with the GH3 domain (PF03321)-encoding region shown in orange. The target sites (red vertical rectangles) of two designed single-guide RNAs (sgRNAs) were located on the third exon. The red and green letters represent the target sequences and the protospacer adjacent motif (PAM) sites, respectively. **(B)** A total of six mutated alleles were identified, from which two alleles, *Osgh3.2-1* and *Osgh3.2-2*, were chosen to further obtain their homozygotes. **(C)** The deduced proteins of wild-type *OsGH3.2* and two mutated alleles *via* Pfam (http://pfam.xfam.org) were shown.

Supplementary Figure 4 *Osgh3.2* mutants showed altered root morphology. Rice roots of wild-type, *Osgh3.2-1* and *Osgh3.2-2* after hydroponic culture **(A)** or inoculation by *R. irregularis* **(C)** for 1 and 2 weeks. Scale bars, 1 cm. **(B,D)** Maximum length of crown roots (CRs), number of large lateral roots (LLRs) and number of CRs were compared between wild type rice and the *Osgh3.2* mutants under two cultivation conditions, respectively. Data are individual values from 4 to 5 biological replicates. The horizontal lines indicate the mean values. Different letters indicate statistically significant differences (Student’s *t*-test, *P* < 0.05).

Supplementary Figure 5 *Osgh3.2* mutants showed higher free IAA and tryptophan levels. **(A,B)** Free IAA and tryptophan (the IAA synthetic precursor) levels were measured in roots of both wild-type rice and *Osgh3.2-1* mutant after hydroponic culture, respectively. Data are individual values from three biological replicates and the horizontal line shows the mean value. Statistical analysis was performed with Student’s *t*-test. Different letters indicate statistically significant differences (*P* < 0.05).

Supplementary Figure 6 *Osgh3.2* mutants exhibited altered panicle architecture and longer grain length. **(A,B)** Images of mature panicles and grains of wild-type rice and the *Osgh3.2* mutants. Scale bars, 1 cm. **(C)** Statistical analysis of panicle length, primary panicle branches and secondary panicle branches among wild -type and *Osgh3.2* mutants, six biological replicates were included. **(D)** Statistical analysis of grain length, grain width and grain thickness among wild type and *Osgh3.2* mutants. Data are individual values from six biological replicates; the horizontal lines indicate the mean values. Different letters indicate statistically significant differences (Student’s *t*-test, *P* < 0.05).

Supplementary Figure 7 An exemplar image of rice root fragment colonized by *R. irregularis* after stained with ink-vinegar solution. The image showed how the longitudinal lengths of arbuscules and cells were measured. Scale bar = 50 μm.

Supplementary Figure 8 Arbuscule populations at 7- and 9-wpi by *R. irregularis* in roots of wild-type rice and the *Osgh3.2* mutants. **(A,B)** The longitudinal lengths of 300 arbuscules from three rice individuals were measured (100 arbuscules per individual) at 7- and 9-wpi, respectively. Arbuscules were classified into three categories according to the longitudinal length, small/degenerate (<30 μm), middle (30–50 μm), and large (>50 μm). Data represent means ± standard deviation (SD). Different letters indicate significant differences (Student’s *t*-test, *P* < 0.05).

Supplementary Table 1 The 250 *GH3* genes identified from 26 surveyed plant genomes.

Supplementary Table 2 The primers used in this study.
