# Supplementary figures and images for: Dual Roles of OsGH3.2 in Modulating Rice Root Morphology and Affecting Arbuscular Mycorrhizal Symbiosis
Source: Front Plant Sci. 2022 Apr 11;13:853435. doi: 10.3389/fpls.2022.853435 (PMC9037295; doi:10.3389/fpls.2022.853435)

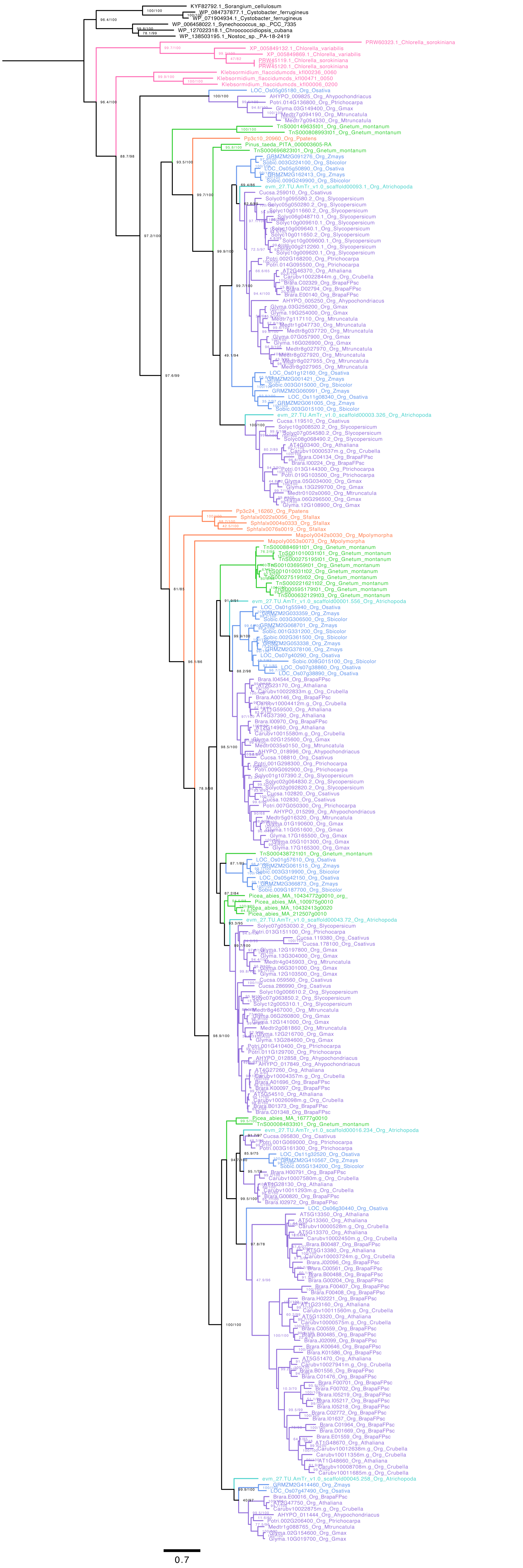

Supplement: Supplementary Figure 1 — A phylogenetic tree of plant GH3 family containing 250 genes identified from 22 plant species. The phylogeny was reconstructed using a maximum-likelihood method under the JTT + I + G4 model via IQ-TREE (Trifinopoulos et al., 2016). The robustness of internal branches was evaluated by calculating the Shimodaira–Hasegawa approximate likelihood ratio test (SH-aLRT) and by performing 1000 ultrafast bootstrap replicates. Six bacterial GH3 genes were designated as the outgroup. The bar represents number of nucleotide substitutions per site. [file Image_1.JPEG]

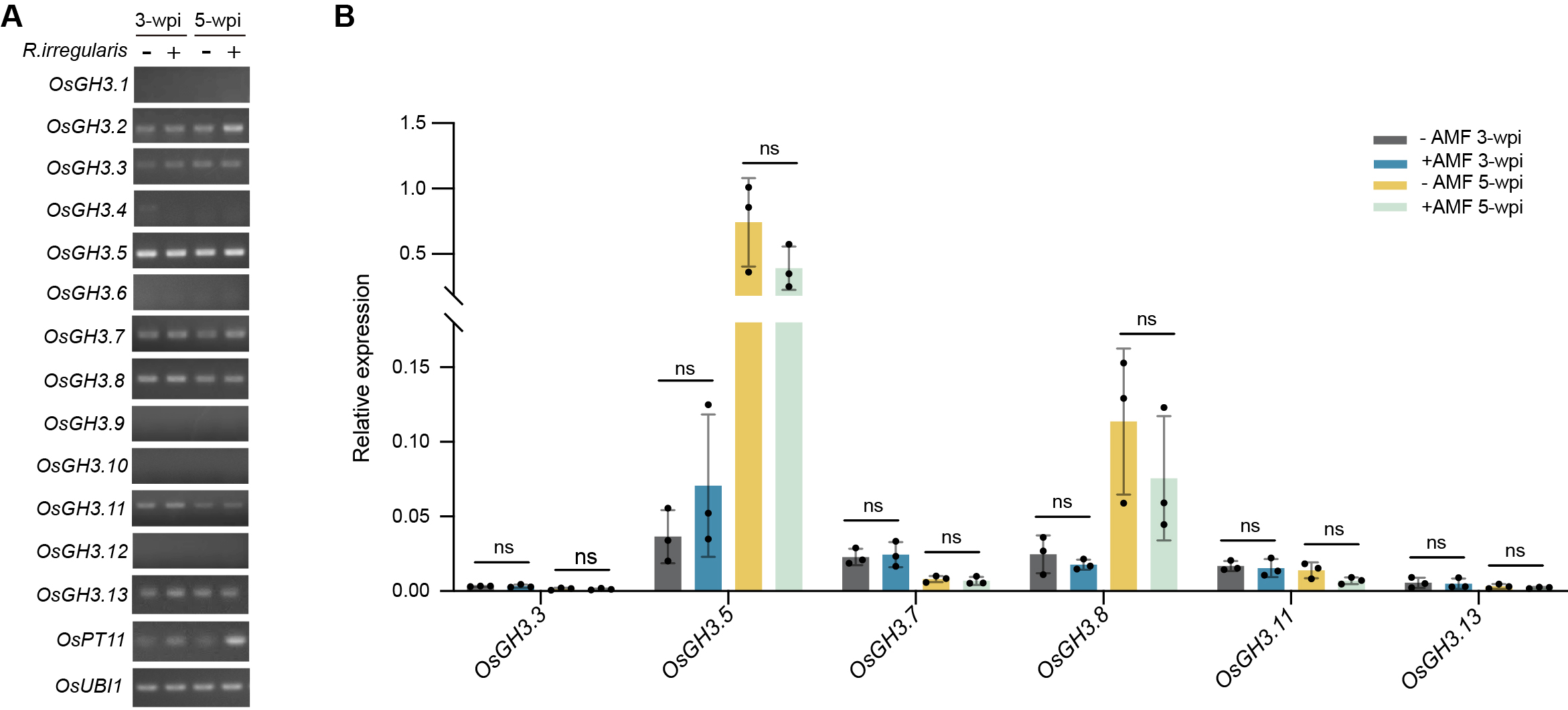

Supplement: Supplementary Figure 2 — The expression patterns of 13 OsGH3 genes in rice roots during AM symbiosis. The transcript abundances of 13 OsGH3 genes in rice roots at 3- and 5-wpi either by mock solution (–) or by R. irregularis (+) were measured by RT-PCR first. (A) Apart from OsGH3.2, six OsGH3 genes appear to be expressed in rice roots, including OsGH3.3, OsGH3.5, OsGH3.7, OsGH3.8, OsGH3.11, and OsGH3.13. (B) Relative expression levels of the six OsGH3 genes were further measured by quantitative RT-PCR. The gene expression values were normalized to the rice housekeeping gene ubiquitin 1 (OsUBI1). Data represent mean ± standard deviations (SD) of three biological replicates. Statistical analysis was performed with Student’s t-test (ns, not significant). [file Image_2.JPEG]

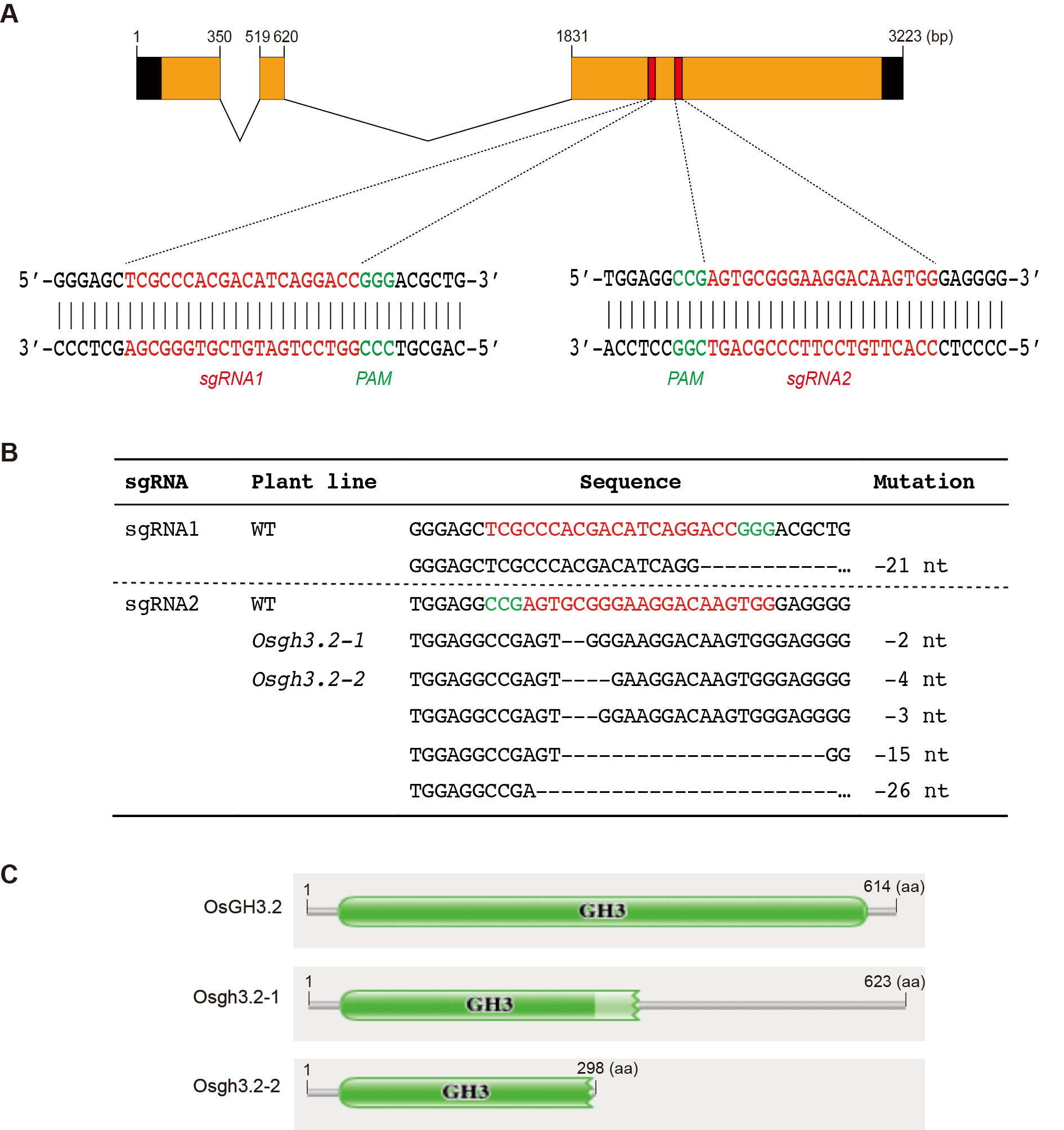

Supplement: Supplementary Figure 3 — CRISPR/Cas9-mediated OsGH3.2 gene mutagenesis in rice. (A) The OsGH3.2 gene has three exons, with the GH3 domain (PF03321)-encoding region shown in orange. The target sites (red vertical rectangles) of two designed single-guide RNAs (sgRNAs) were located on the third exon. The red and green letters represent the target sequences and the protospacer adjacent motif (PAM) sites, respectively. (B) A total of six mutated alleles were identified, from which two alleles, Osgh3.2-1 and Osgh3.2-2, were chosen to further obtain their homozygotes. (C) The deduced proteins of wild-type OsGH3.2 and two mutated alleles via Pfam (http://pfam.xfam.org) were shown. [file Image_3.JPEG]

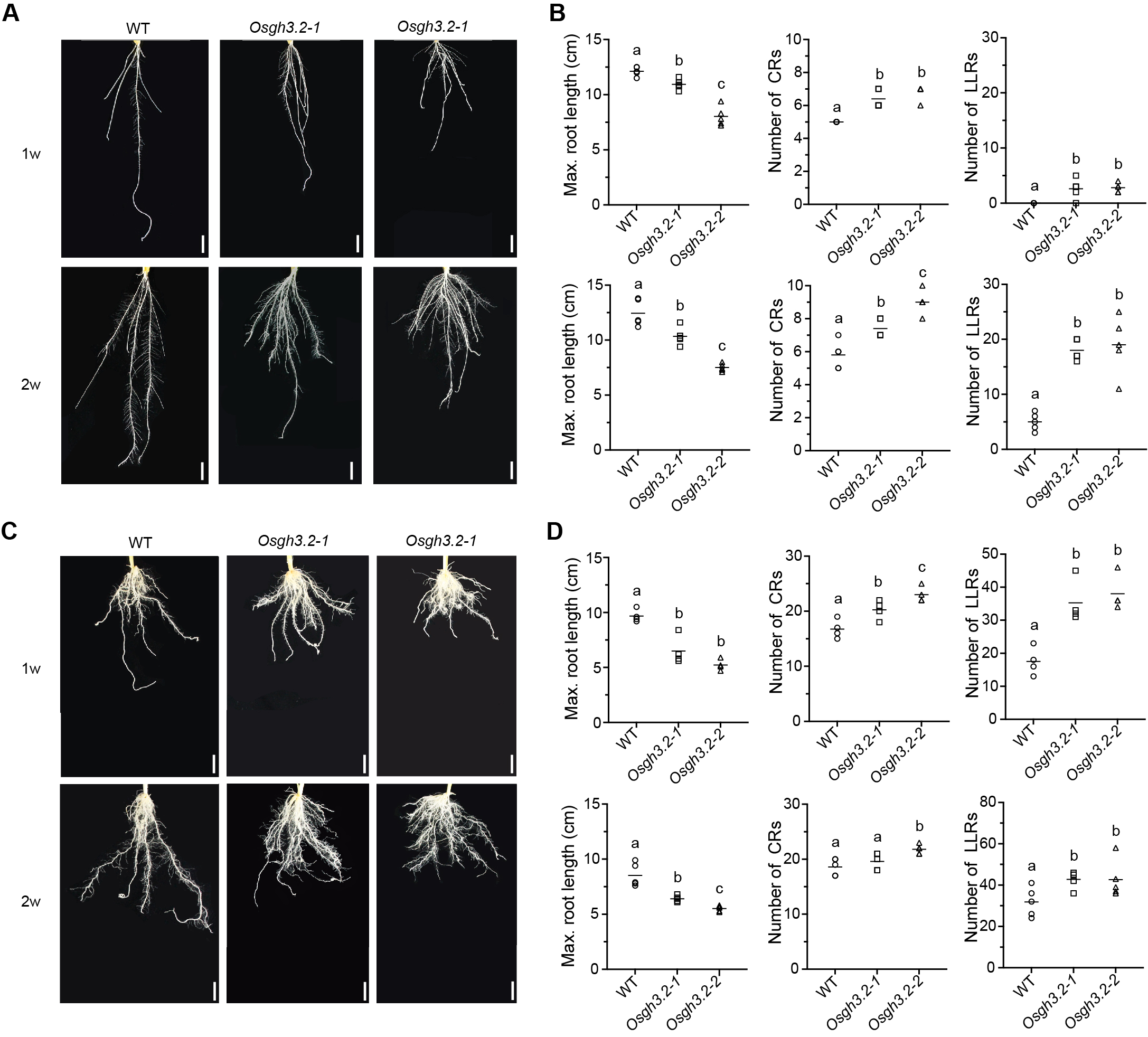

Supplement: Supplementary Figure 4 — Osgh3.2 mutants showed altered root morphology. Rice roots of wild-type, Osgh3.2-1 and Osgh3.2-2 after hydroponic culture (A) or inoculation by R. irregularis (C) for 1 and 2 weeks. Scale bars, 1 cm. (B,D) Maximum length of crown roots (CRs), number of large lateral roots (LLRs) and number of CRs were compared between wild type rice and the Osgh3.2 mutants under two cultivation conditions, respectively. Data are individual values from 4 to 5 biological replicates. The horizontal lines indicate the mean values. Different letters indicate statistically significant differences (Student’s t-test, P < 0.05). [file Image_4.JPEG]

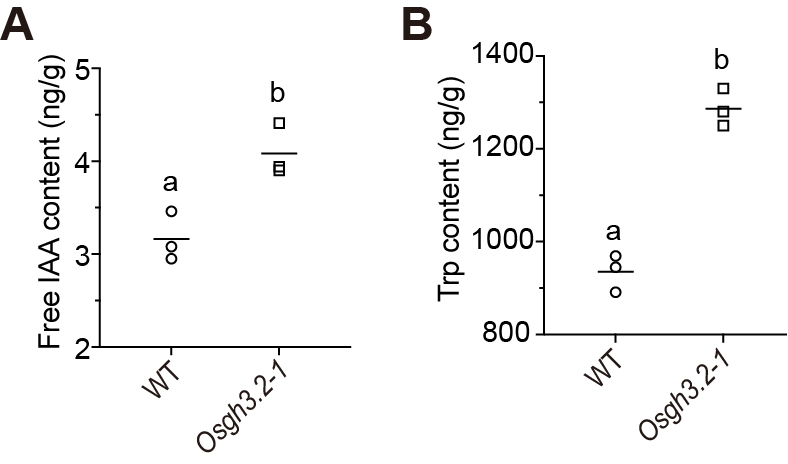

Supplement: Supplementary Figure 5 — Osgh3.2 mutants showed higher free IAA and tryptophan levels. (A,B) Free IAA and tryptophan (the IAA synthetic precursor) levels were measured in roots of both wild-type rice and Osgh3.2-1 mutant after hydroponic culture, respectively. Data are individual values from three biological replicates and the horizontal line shows the mean value. Statistical analysis was performed with Student’s t-test. Different letters indicate statistically significant differences (P < 0.05). [file Image_5.JPEG]

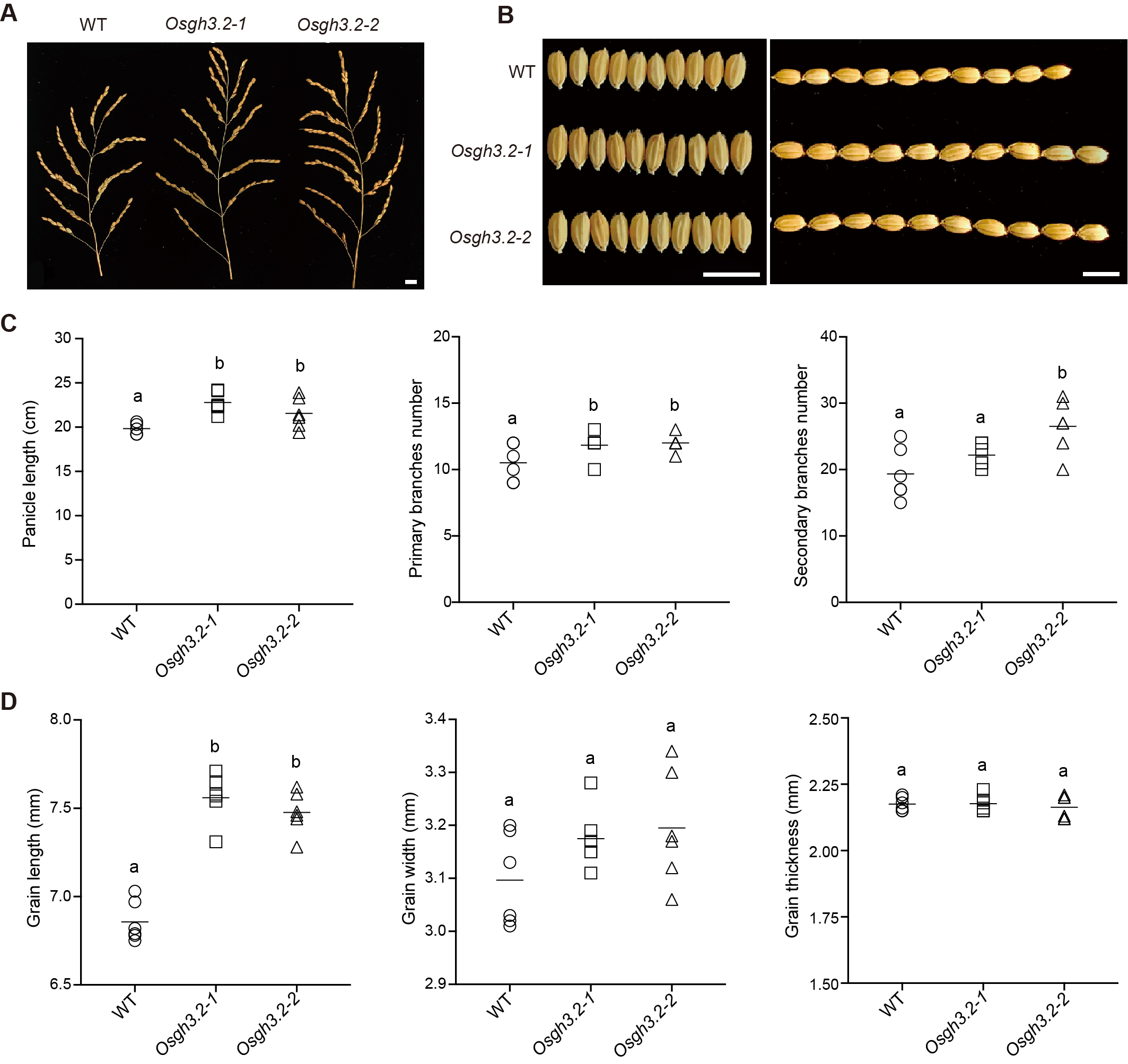

Supplement: Supplementary Figure 6 — Osgh3.2 mutants exhibited altered panicle architecture and longer grain length. (A,B) Images of mature panicles and grains of wild-type rice and the Osgh3.2 mutants. Scale bars, 1 cm. (C) Statistical analysis of panicle length, primary panicle branches and secondary panicle branches among wild -type and Osgh3.2 mutants, six biological replicates were included. (D) Statistical analysis of grain length, grain width and grain thickness among wild type and Osgh3.2 mutants. Data are individual values from six biological replicates; the horizontal lines indicate the mean values. Different letters indicate statistically significant differences (Student’s t-test, P < 0.05). [file Image_6.JPEG]

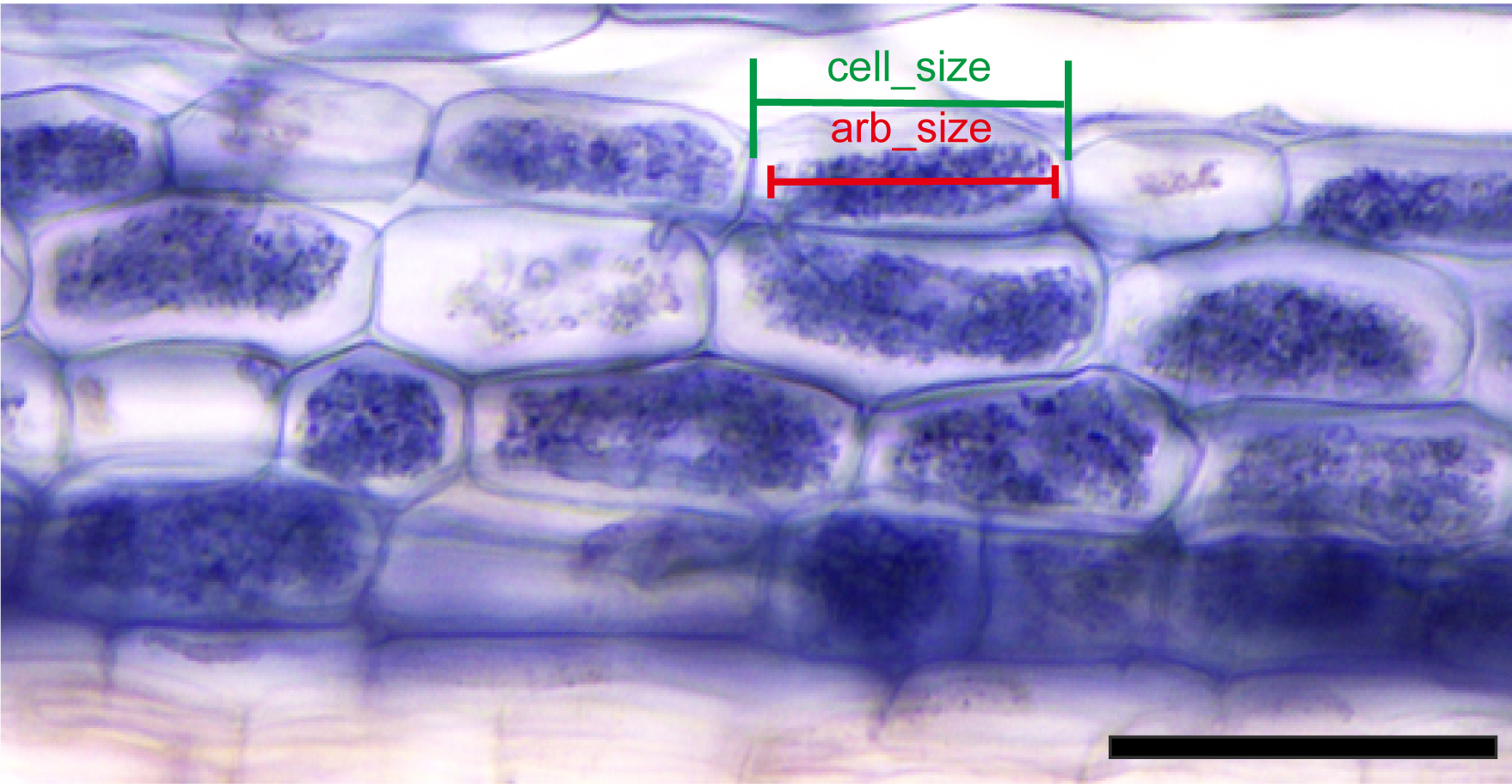

Supplement: Supplementary Figure 7 — An exemplar image of rice root fragment colonized by R. irregularis after stained with ink-vinegar solution. The image showed how the longitudinal lengths of arbuscules and cells were measured. Scale bar = 50 μm. [file Image_7.JPEG]

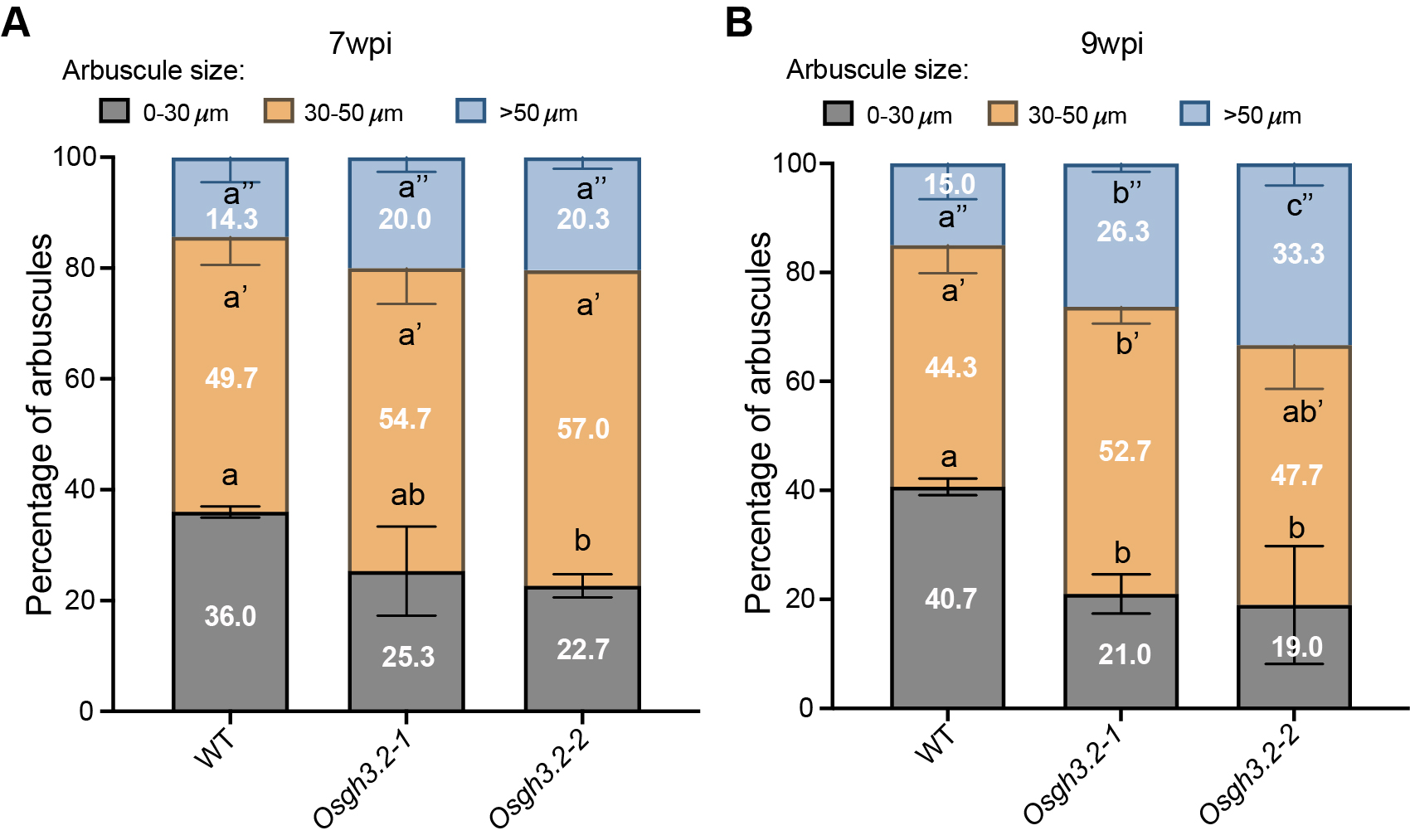

Supplement: Supplementary Figure 8 — Arbuscule populations at 7- and 9-wpi by R. irregularis in roots of wild-type rice and the Osgh3.2 mutants. (A,B) The longitudinal lengths of 300 arbuscules from three rice individuals were measured (100 arbuscules per individual) at 7- and 9-wpi, respectively. Arbuscules were classified into three categories according to the longitudinal length, small/degenerate (<30 μm), middle (30–50 μm), and large (>50 μm). Data represent means ± standard deviation (SD). Different letters indicate significant differences (Student’s t-test, P < 0.05). [file Image_8.JPEG]
