# Supplementary material for: Dual Roles of OsGH3.2 in Modulating Rice Root Morphology and Affecting Arbuscular Mycorrhizal Symbiosis
Source: Front Plant Sci. 2022 Apr 11;13:853435. doi: 10.3389/fpls.2022.853435 (PMC9037295; doi:10.3389/fpls.2022.853435)
Supplement: Supplementary Table 1 — The 250 GH3 genes identified from 26 surveyed plant genomes. [file Table_1.DOCX]

**Table S1. The 250 *GH3* genes identified from 26 surveyed plant genomes.**

| **Organism** | **Version/ reference** | **Website** | **Number** | **Gene** |
| --- | --- | --- | --- | --- |
| *Chlorella variabills* | v1.0 | https://www.ncbi.nlm.nih.gov/genome/694?genome_assembly_id=30556 | 2 | XP_005849869.1  XP_005849132.1 |
| *Chlorella sorokiniana* | v1.0 | https://www.ncbi.nlm.nih.gov/genome/31394?genome_assembly_id=382030 | 3 | PRW45119.1  PRW45120.1  PRW60323.1 |
| *Chlamydomonas reinhardtii* | v5.5 | https://phytozome.jgi.doe.gov/pz/portal.html | 0 | -- |
| *Ostreococcus lucimarinus* | v2.0 | https://phytozome.jgi.doe.gov/pz/portal.html | 0 | -- |
| *Chlorokybus atmophyticus* | v1.0 | https://www.ncbi.nlm.nih.gov/genome/10735?genome_assembly_id=712935 | 0 | -- |
| *Mesostigma viride* | v1.0 | https://www.ncbi.nlm.nih.gov/genome/2956?genome_assembly_id=748600 | 0 | -- |
| *Klebsormidium flaccidum* | v1.1 | http://www.plantmorphogenesis.bio.titech.ac.jp/~algae_genome_project/klebsormidium/ | 3 | kfl00471_0050  kfl00236_0060  kfl00006_0200 |
| *Marchantia polymorpha* | v3.1 | https://phytozome.jgi.doe.gov/pz/portal.html | 2 | Mapoly0042s0030  Mapoly0053s0073 |
| *Physcomitrella patens* | v3.3 | https://phytozome.jgi.doe.gov/pz/portal.html | 2 | Pp3c10_20960 (PpGH3.2)  Pp3c24_16260 (PpGH3.1) |
| *Sphagnum fallax* | v0.5 | https://phytozome.jgi.doe.gov/pz/portal.html | 3 | Sphfalx0004s0333  Sphfalx0022s0056  Sphfalx0076s0019 |
| *Gnetum montanum* | Wan et al., Nature Plants 2018, 4: 82–89 | https://datadryad.org | 14 | TnS000084833t01  TnS000438721t01  TnS000696823t01  TnS001036959t01  TnS001010031t02  TnS000275195t02  TnS000595179t01  TnS000884691t01  TnS000221621t02  TnS000632129t03  TnS001010031t01  TnS000275195t01  TnS000149635t01  TnS000808993t01 |
| *Picea abies* | Nystedt et al., Nature, 2013, 497(7451):579-84 | https://www.ncbi.nlm.nih.gov/genome/11155?genome_assembly_id=367163 | 5 | 10434772g0010  10432413g0020  100975g0010  16777g0010  212507g0010 |
| *Pinus taeda* | v2.0 | https://www.ncbi.nlm.nih.gov/genome/11027?genome_assembly_id=301789 | 1 | 000003605 |
| *Amborella trichopoda* | v1.0 | https://phytozome.jgi.doe.gov/pz/portal.html | 6 | Ambtr00001.556  Ambtr00003.326  Ambtr00016.234  Ambtr00043.72  Ambtr00045.258  Ambtr00093.1 |
| *Oryza sativa* | v7_JGI | https://phytozome.jgi.doe.gov/pz/portal.html | 13 | Orysa01g57610 (OsGH3.1)  Orysa01g55940 (OsGH3.2)  Orysa01g12160 (OsGH3.3)  Orysa05g42150 (OsGH3.4)  Orysa05g50890 (OsGH3.5)  Orysa05g05180 (OsGH3.6)  Orysa06g30440 (OsGH3.7)  Orysa07g40290 (OsGH3.8)  Orysa07g38890 (OsGH3.9)  Orysa07g38860 (OsGH3.10)  Orysa07g47490 (OsGH3.11)  Orysa11g08340 (OsGH3.12)  Orysa11g32510+520 (OsGH3.13) |
| *Sorghum bicolor* | v3.1.1 | https://phytozome.jgi.doe.gov/pz/portal.html | 11 | Sorbi001G331200  Sorbi002G361500  Sorbi003G015000  Sorbi003G015100  Sorbi003G224100  Sorbi003G306500  Sorbi003G319900  Sorbi005G134200  Sorbi008G015100  Sorbi009G187700  Sorbi009G249900 |
| *Zea mays* | Ensembl-18 | https://phytozome.jgi.doe.gov/pz/portal.html | 13 | GRMZM2G001421  GRMZM2G033359  GRMZM2G053338  GRMZM2G060991  GRMZM2G061005  GRMZM2G061515  GRMZM2G068701  GRMZM2G091276  GRMZM2G162413  GRMZM2G366873  GRMZM2G378106  GRMZM2G410567  GRMZM2G414460 |
| *Amaranthus hypochondriacus* | v1.0 | https://phytozome.jgi.doe.gov/pz/portal.html | 7 | AHYPO_005250  AHYPO_009825  AHYPO_011444  AHYPO_012858  AHYPO_015299  AHYPO_017849  AHYPO_018996 |
| *Solanum lycopersicum* | iTAG2.4 | https://phytozome.jgi.doe.gov/pz/portal.html | 20 | Solly00g212260.1  Solly01g095580.2 (SlGH3.1)  Solly01g107390.2 (SlGH3.2)  Solly02g064830.2 (SlGH3.3)  Solly02g092820.2 (SlGH3.4)  Solly05g050280.2 (SlGH3.5)  Solly06g048710.1 (SlGH3.6)  Solly07g053030.2 (SlGH3.7)  Solly07g054580.2 (SlGH3.8)  Solly07g063850.2 (SlGH3.9)  Solly08g068490.2  Solly10g006610.2  Solly10g008520.2 (SlGH3.10)  Solly10g009600.1 (SlGH3.11)  Solly10g009610.1 (SlGH3.12)  Solly10g009620.1 (SlGH3.13)  Solly10g009640.1  Solly10g011650.2  Solly10g011660.2 (SlGH3.14)  Solly12g005310.1 (SlGH3.15) |
| *Brassica rapa* | v1.2 | https://phytozome.jgi.doe.gov/pz/portal.html | 41 | Brara.A00146  Brara.A01696  Brara.B00485  Brara.B00487  Brara.B00488  Brara.B01373  Brara.B01556  Brara.C00559  Brara.C00561  Brara.C01348  Brara.C01476  Brara.C01964  Brara.C02329  Brara.C02772  Brara.C04134  Brara.D01669  Brara.D02794  Brara.E00016  Brara.E00140  Brara.E01559  Brara.F00407  Brara.F00408  Brara.F00701  Brara.F00702  Brara.G00204  Brara.G00820  Brara.H00791  Brara.H02221  Brara.I00224  Brara.I00970  Brara.I01637  Brara.I02972  Brara.I04544  Brara.I05217  Brara.I05218  Brara.I05219  Brara.J02096  Brara.J02099  Brara.K00097  Brara.K00646  Brara.K01586 |
| *Capsella rubella* | v1.0 | https://phytozome.jgi.doe.gov/pz/portal.html | 20 | Carubv10000528  Carubv10000537  Carubv10000575  Carubv10002450  Carubv10003724  Carubv10004357  Carubv10004412  Carubv10007580  Carubv10008708  Carubv10011293  Carubv10011356  Carubv10011560  Carubv10011685  Carubv10012638  Carubv10015580  Carubv10022833  Carubv10022844  Carubv10022875  Carubv10026098  Carubv10027941 |
| *Arabidopsis thaliana* | TAIR10 | https://phytozome.jgi.doe.gov/pz/portal.html | 19 | Arath2G14960 (AtGH3.1)  Arath4G37390 (AtGH3.2)  Arath2G23170 (AtGH3.3)  Arath1G59500 (AtGH3.4)  Arath4G27260 (AtGH3.5)  Arath5G54510 (AtGH3.6)  Arath1G23160 (AtGH3.7)  Arath5G51470 (AtGH3.8)  Arath2G47750 (AtGH3.9)  Arath4G03400 (AtGH3.10)  Arath2G46370 (AtGH3.11)  Arath5G13320 (AtGH3.12)  Arath5G13350 (AtGH3.13)  Arath5G13360 (AtGH3.14)  Arath5G13370 (AtGH3.15)  Arath5G13380 (AtGH3.16)  Arath1G28130 (AtGH3.17)  Arath1G48670 (AtGH3.18)  Arath1G48660 (AtGH3.19) |
| *Populus trichocarpa* | v3.0 | https://phytozome.jgi.doe.gov/pz/portal.html | 14 | Poptr001G069000  Poptr001G298300  Poptr001G410400  Poptr002G168200  Poptr002G206400  Poptr003G161300  Poptr007G050300  Poptr009G092900  Poptr011G129700  Poptr013G144300  Poptr013G151100  Poptr014G095500  Poptr014G136800  Poptr019G103500 |
| *Cucumis sativus* | v1.0 | https://phytozome.jgi.doe.gov/pz/portal.html | 10 | Cucsa.059560  Cucsa.095830  Cucsa.102820  Cucsa.102830  Cucsa.108810  Cucsa.119380  Cucsa.119510  Cucsa.178100  Cucsa.259010  Cucsa.286990 |
| *Glycine max* | Wm82.a2.v1 | https://phytozome.jgi.doe.gov/pz/portal.html | 25 | Glyma.01G190600  Glyma.02G125600  Glyma.02G154600  Glyma.03G149400  Glyma.03G256200  Glyma.05G034000  Glyma.05G101300  Glyma.06G260800  Glyma.06G296500  Glyma.06G301000  Glyma.07G057900  Glyma.10G019700  Glyma.11G051600  Glyma.12G103500  Glyma.12G108900  Glyma.12G141000  Glyma.12G197800  Glyma.12G216700  Glyma.13G284600  Glyma.13G299700  Glyma.13G304000  Glyma.16G026900  Glyma.17G165300  Glyma.17G165500  Glyma.19G254000 |
| *Medicago truncatula* | Mt4.0v1 | https://phytozome.jgi.doe.gov/pz/portal.html | 16 | Medtr0035s0150 (MtGH3.16)  Medtr0102s0060 (MtGH3.17)  Medtr1g047730 (MtGH3.1)  Medtr1g088765 (MtGH3.2)  Medtr2g081860 (MtGH3.3)  Medtr4g045903 (MtGH3.5)  Medtr5g016320 (MtGH3.6)  Medtr7g094190 (MtGH3.7)  Medtr7g094330 (MtGH3.8)  Medtr7g117110 (MtGH3.9)  Medtr8g027920 (MtGH3.10)  Medtr8g027955 (MtGH3.11)  Medtr8g027965 (MtGH3.12)  Medtr8g027970 (MtGH3.13)  Medtr8g037720 (MtGH3.14)  Medtr8g467000 (MtGH3.15) |
