# Supplementary material for: Dual Roles of OsGH3.2 in Modulating Rice Root Morphology and Affecting Arbuscular Mycorrhizal Symbiosis
Source: Front Plant Sci. 2022 Apr 11;13:853435. doi: 10.3389/fpls.2022.853435 (PMC9037295; doi:10.3389/fpls.2022.853435)
Supplement: Supplementary Table 2 — The primers used in this study. [file Table_2.DOCX]

**Table S2. The primers used in this study.**

| Primer names | Primer sequences (5’-3’) |
| --- | --- |
| For *OsGH3.2* editing |  |
| *OsGH3.2-spacer1-F* | ggcaTCGCCCACGACATCAGGACC |
| *OsGH3.2-spacer1-R* | aaacGGTCCTGATGTCGTGGGCGA |
| *OsGH3.2-spacer2-F* | ggcaCCACTTGTCCTTCCCGCACT |
| *OsGH3.2-spacer2-R* | aaacAGTGCGGGAAGGACAAGTGG |
| *pRGEB31-Cas9-F* | CAGATGTGCAGTCAGGGACC |
| *pRGEB31-Cas9-R* | CATGCACGCGCTAAAAACGG |
| *OsGH3.2 edit-F* | AATCCGGCATGCAGAACTCA |
| *OsGH3.2 edit-R* | AGAACTTGAGCGTGGGGATG |
|  |  |
| For *OsGH3.2* promoter |  |
| *OsGH3.2pro-HindIII-F* | cccaagcttTCCATTCCCTGTACGGCTGA |
| *OsGH3.2pro-BamHI-R* | cgcggatccCCCTAATTAGTTGGCGTTGCT |
| *OsGH3.2pro-F* | AAGCAGCAACAAAACACCCC |
| *PBI101.1-GUS-R* | CCTGCCCAACCTTTCGGTAT |
|  |  |
| For qRT-PCR |  |
| *qOsUBI-F* | TGCACCCTAGGGCTGTCAAC |
| *qOsUBI-R* | GACGCTCTAGTTCTTGATCTTCTTC |
| *qOsGH3.1-F* | ATCGCCGACGAGATGAACAG |
| *qOsGH3.1-R* | GGCTCCGGTAGTAGCTTGTG |
| *qOsGH3.2-F* | AGGAAGCTAATGCCGACGAT |
| *qOsGH3.2-R* | CGCTCTTGTAGTAGCTGGTCA |
| *qOsGH3.3-F* | CCACCTTCGCAGGGCTTTA |
| *qOsGH3.3-R* | TGCTCGCTGTTCTTGTCGAT |
| *qOsGH3.4-F* | TCAACGAGTGTTAGTTCAGGGT |
| *qOsGH3.4-R* | GGGTTTGGAACAACAAAAGCG |
| *qOsGH3.5-F* | GGAGACAGAAAACTCCGCCT |
| *qOsGH3.5-R* | GGAGCTCAGGTGTGGAGTTG |
| *qOsGH3.6-F* | GCCTGAGGAAACCACCTACG |
| *qOsGH3.6-R* | GGTCACGAACTGAAGCCGAG |
| *qOsGH3.7-F* | ATCCACCATCCCATGTGTCA |
| *qOsGH3.7-R* | CCCGGTACCTGTAAAGACCTG |
| *qOsGH3.8-F* | TGTATGTGCCTGGGCTTGAC |
| *qOsGH3.8-R* | TCGTGTAGTTGTGGTACGGG |
| *qOsGH3.9-F* | CGGCCTACTTCGAGTTCCTC |
| *qOsGH3.9-R* | GCCTCGTCGGTCTTGTCTAA |
| *qOsGH3.10-F* | TTGAACGTGTACGTGCCTGG |
| *qOsGH3.10-R* | TGTAGGCGTAGGCCCTCTT |
| *qOsGH3.11-F* | AACAAGTATATATCAGGCCTTGGTG |
| *qOsGH3.11-R* | AAAGACTGCCCCGATACGAA |
| *qOsGH3.12-F* | ATCTCCCTAAGCTCCGGCAC |
| *qOsGH3.12-R* | CATGAACTGCAACACCCTGC |
| *qOsGH3.13-F* | GCGTCGTCGGAGTCCTACTT |
| *qOsGH3.13-R* | GCCGTCCTTCTCCACCTTGAT |
| *qOsPT11-F* | GAGAAGTTCCCTGCTTCAAGCA |
| *qOsPT11-R* | CATATCCCAGATGAGCGTATCATG |
| *qOsD17-F* | CGCACTACAAGATCGACCCG |
| *qOsD17-R* | GTGGATCATGAGGTGGTCCG |
| *qOsAFB2-F* | CGTTTGTCAATATCTGGTC |
| *qOsAFB2-R* | GACATCCAAAGGGATCGCA |
